# Dietary restriction mitigates the age-associated decline in mouse B cell receptor repertoire diversity

**DOI:** 10.1016/j.celrep.2023.112722

**Published:** 2023-06-28

**Authors:** Carolina Monzó, Lisonia Gkioni, Andreas Beyer, Dario Riccardo Valenzano, Sebastian Grönke, Linda Partridge

**Affiliations:** 1 Department Biological Mechanisms of Ageing, Max Planck Institute for Biology of Ageing, 50931 Cologne, North Rhine Westphalia, Germany; 2 Cologne Excellence Cluster on Cellular Stress Responses in Age-Associated Diseases (CECAD), Faculty of Medicine and Faculty of Mathematics and Natural Sciences, University of Cologne, 50931 Cologne, Germany; 3 Microbiome-Host Interactions in Ageing Group, Max Planck Institute for Biology of Ageing, 50931 Cologne, North Rhine Westphalia, Germany; 4 Evolutionary Biology/Microbiome-Host Interactions in Aging Group: Fritz Lipmann Institute - Leibniz Institute on Aging, 07745 Jena, Thuringia, Germany; 5 Genetics, Evolution & Environment Group, Institute of Healthy Ageing, University College London, London WC1E 6BT, UK

**Keywords:** adaptive immunity, B cell receptor repertoire, dietary restriction, late onset, aging, mice

## Abstract

Aging impairs the capacity to respond to novel antigens, reducing immune protection against pathogens and vaccine efficacy. Dietary restriction (DR) extends life- and health span in diverse animals. However, little is known about the capacity of DR to combat the decline in immune function. Here, we study the changes in B cell receptor (BCR) repertoire during aging in DR and control mice. By sequencing the variable region of the BCR heavy chain in the spleen, we show that DR preserves diversity and attenuates the increase in clonal expansions throughout aging. Remarkably, mice starting DR in mid-life have repertoire diversity and clonal expansion rates indistinguishable from chronic DR mice. In contrast, in the intestine, these traits are unaffected by either age or DR. Reduced within-individual B cell repertoire diversity and increased clonal expansions are correlated with higher morbidity, suggesting a potential contribution of B cell repertoire dynamics to health during aging.

## Highlights

• DR preserves B cell receptor diversity during aging in the mouse spleen
• Mid-life onset of DR is sufficient to improve B cell receptor diversity in the spleen
• Despite changes in the microbiome, BCR diversity is not affected by DR in the ileum
• DR-dependent B cell repertoire metrics are inversely correlated with morbidity

## Graphical abstract

**Figure:**
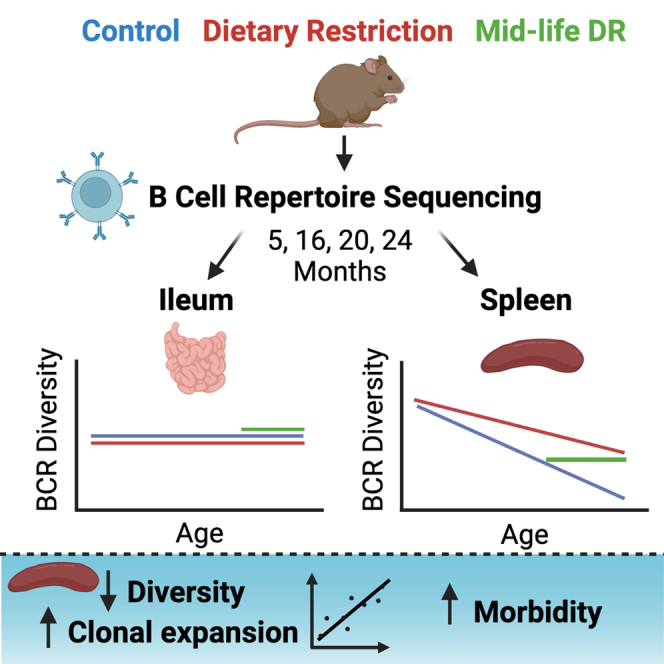

## Teaser

Monzó et al. study how aging impacts the immune system and if dietary restriction (DR) can counteract it. They focus on B cells and discover that DR maintains B cell diversity and decreases clonal expansions, even when initiated in mid-life, implicating DR as potential treatment to improve immunity at old age.

## Introduction

During aging, the adaptive immune system undergoes a profound functional dysregulation^1^^,^^2^^,^^3^ resulting in impaired responses to pathogens and vaccination.^4^^,^^5^^,^^6^ In humans and mice, naive B cells become displaced by antigen-experienced (memory) B cells, resulting in loss of B cell receptor (BCR) repertoire diversity and antigen specificity.^6^^,^^7^^,^^8^^,^^9^^,^^10^^,^^11^ Low diversity in BCRs is associated with poor antigen-recognition capacity and vaccination response.^8^^,^^12^^,^^13^ Increased clonal expansions and positive selection of high-affinity B cells further impair immune function and are also associated with poor health and frailty.^14^^,^^15^^,^^16^ It is therefore important to discover whether anti-aging interventions can ameliorate or reverse the loss of BCR diversity and clonal expansions during aging.

Dietary restriction (DR), reduced nutrient intake without malnutrition, is a highly effective intervention to extend life span and health span in multiple animal species, and it can ameliorate morbidity and mortality in old age.^17^^,^^18^^,^^19^^,^^20^^,^^21^ DR induces changes in the context of T cell-mediated responses, hematopoietic stem cells, and B cell maturation stages.^2^^,^^22^^,^^23^ More specifically, DR maintains the production of naive T cells and T cell receptor repertoire diversity in aged non-human primates.^2^^,^^22^^,^^23^ DR also increases B cell maturation through a decline in the total B cell population and an increased recirculation of mature B cells in male mice.^23^ However, it remains unknown how DR affects the BCR repertoire diversity. Detailed documentation of the changes in the aging BCR repertoire diversity under DR can be instrumental in deepening our understanding of DR implementation as a means to confer reduced susceptibility to infections and greater vaccine efficacy.

Initiation of DR at older ages can improve subsequent health, avoiding the need for long-term DR feeding, potentially of translational relevance to human.^24^^,^^25^^,^^26^ However, how DR starting either early or later in adulthood affects the diversity of the BCR repertoire is unexplored.

It is therefore important to establish whether later-onset DR can ameliorate the age-related decline in BCR repertoire diversity and clonal expansions. We have thus examined the effects of DR initiated at either 3 or 16 months on these traits.

In the present study, we performed BCR sequencing^27^ on spleen and ileum of female mice aged 5, 16, 20, and 24 months. We found that, in the spleen, DR initiated in young adults maintained both the BCR repertoire diversity and lower clonal expansions during aging. Furthermore, mice subjected to DR starting at 16 months had spleen BCR diversity and clonal expansion rates indistinguishable from those with chronic DR, suggesting an acute effect of DR. In contrast to the spleen, the BCR repertoire of the ileum showed only limited changes with age and in response to either early- or later-onset DR. However, in the ileum of old mice, DR initiated in young adults increased somatic hypermutation frequency, which is the mechanism for affinity maturation of the BCR repertoire in response to antigen exposure that diversifies the repertoire,^8^^,^^28^ suggesting an improved capacity for antigen binding under DR. Within-individual B cell repertoire diversity and clonal expansions in the spleen were inversely correlated with the morbidity of individual mice, suggesting a contribution of these traits to health during aging.

## Results

The BCR consists of two identical heavy chains (immunoglobulin [Ig] H) coded by the *Igh* gene, and two light chains (IgL). The heavy chains, which are sufficient to identify B cell clonal relationships,^29^ have a variable domain, encompassed by a combination of *IghV*, *IghD*, and *IghJ* genes, and a constant domain (*IghC*). After antigen identification by the variable domain, *IghC* regions undergo class-switch recombination, where μ (IgM, known for its role in the primary immune response) and δ (IgD, whose role in immune responses is still largely unknown) are substituted by either γ, ε, or α heavy chains, giving rise to isotypes with different effector functions, namely IgG (response against viruses and bacteria), IgE (response to parasites and allergens), and IgA (response to mucosal microbes), respectively.^28^^,^^30^ In this study, we analyzed clonal population structure to understand how BCR repertoire composition is influenced by aging and DR. In addition, we studied individual isotypes to elucidate the potential functional implications associated with their distinct effector roles.

**Figure 1:**
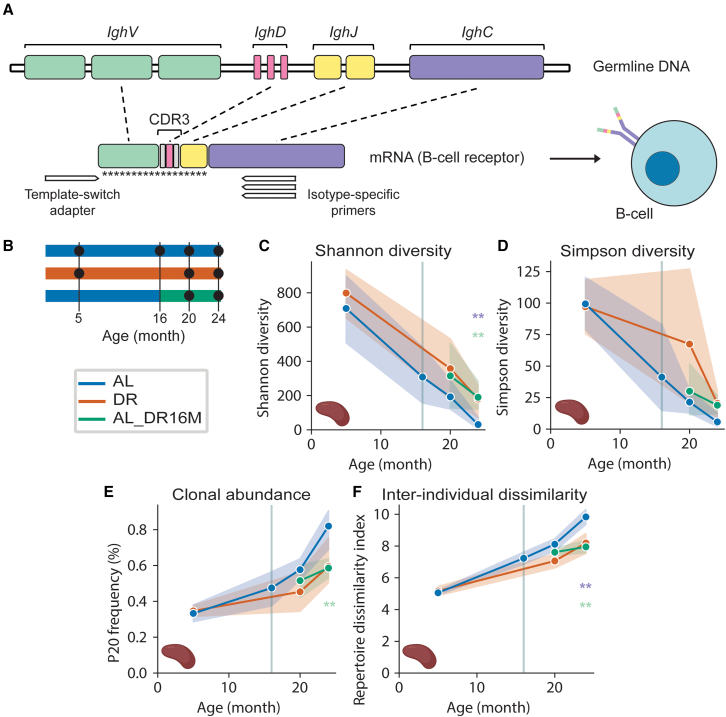
DR slows the age-associated decline of BCR repertoire in the spleen (A) Schematic of IgH heavy-chain gene arrangement of BCRs and location of BCR isotype-specific primers. Asterisks indicate the region modified by somatic hypermutations. (B) Outline of the DR experiment. Black circles indicate time points when spleen and ileum samples were taken. n = 5 female mice per treatment. (C) Shannon within-individual diversity. Significant differences through age (linear regression): AL, p = 0.000001; DR p = 0.0002. Significant differences through age and diet (two-way ANOVA): AL vs. DR age p = 1.11 × 10^−8^; diet p = 0.049; age × diet p = 0.63; AL vs. AL_DR16 age p = 0.058, diet p = 0.061, age × diet p = 0.79; DR vs. AL_DR16 age p = 0.104, diet p = 0.84, age × diet p = 0.767. Significant differences at 24 months of age (Mann-Whitney U test): DR vs. AL, p = 0.004; AL_DR16M vs. AL, p = 0.008. (D) Simpson within-individual diversity. Significant differences through age (linear regression): AL, p = 0.0000003; DR, p = 0.03. Significant differences through age and diet (two-way ANOVA): AL vs. DR age p = 0.000025; diet p = 0.144305; age × diet p = 0.307337; AL vs. AL_DR16 age p = 0.092965; diet p = 0.164725; age × diet p = 0.759599; DR vs. AL_DR16 age p = 0.141316; diet p = 0.272678; age × diet p = 0.335867. Significant differences between 20 and 24 months of age (Mann-Whitney U test): DR p = 0.036. (E) Clonal abundance. Significant differences through age (linear regression): AL p = 0.00002; DR p = 0.02. Significant differences through age and diet (two-way ANOVA): AL vs. DR age p = 0.000003; diet p = 0.030556; age × diet p = 0.048481; AL vs. AL_DR16 age p = 0.004114; diet p = 0.006239; age × diet p = 0.073774; DR vs. AL_DR16 age p = 0.094003; diet p = 0.631426; age × diet p = 0.549253. Significant differences at 24 months of age (Mann-Whitney U test): AL_DR16M vs. AL p = 0.002. Significant differences between 20 and 24 months of age (Mann-Whitney U test): DR p = 0.036. (F) Inter-individual dissimilarity. Significant differences through age (linear regression): AL p = 1.0 × 10^−17^; DR p = 1.0 × 10^−7^. Significant differences through age and diet (two-way ANOVA): AL vs. DR age p = 3.7 × 10^−22^; diet p = 0.00007; age × diet p = 0.0007556679; AL vs. AL_DR16 age p = 0.000033; diet p = 0.000003; age × diet p = 0.002995; DR vs. AL_DR16 age p = 0.005271; diet p = 0.531227; age × diet p = 0.118437. Significant differences at 24 months of age (Mann-Whitney U test): DR vs. AL p = 0.002, AL vs. AL_DR16M p = 2.0 × 10^−4^. Significant differences between baseline AL at 16 months of age and mice at 20 months of age (Mann-Whitney U test): AL vs. AL p = 0.015. Lines correspond to mean and shaded area to 95% confidence intervals. n = 5 female mice per treatment. ^∗∗^p < 0.01 (Mann-Whitney U test) at individual time points represented in light purple for DR vs. AL and light green for AL vs. AL_DR16M. Relevant and significant p values are indicated; for all non-significant p values, see Table S1.

To investigate how aging and DR affect BCR repertoire, we sequenced the variable (*IghV*, *IghD*, *IghJ*) region of the BCR heavy chain (Figure 1A) of wild-type, female C3B6F1 hybrid mice fed *ad libitum* (AL) or subjected to DR from the age of 3 months (DR) (Figure 1B). DR mice received 40% less food than the amount consumed by AL control animals.^31^ To address whether mid-life onset of DR could also affect the BCR repertoire, we included mice where DR was initiated at 16 months of age (AL_DR16M) (Figure 1B). Total RNA was isolated from the spleen and ileum, to capture the systemic or gut-specific profiles, respectively, of five mice per treatment at 5, 20, and 24 months of age (Figure 1B). We limited our analysis to BCR heavy chains, as they are sufficient to identify clonal relationships with high confidence.^29^ BCR clones from the same naive B cell ancestor were defined by sequences sharing the same *IghV* and *IghJ* gene (Figures 1A and S1A–S1D) and having identical amino acid complementarity-determining regions 3 (CDR3) (Figures 1A, S1E, and S1F).^32^^,^^33^^,^^34^ BCR isotypes were identified by a template-switch adapter (i.e., an oligonucleotide that results in a switch of templates by the reverse transcriptase, thereby generating amplified cDNA enriched for full-length sequences) in the 5′ of the *IghV* variable domain and isotype-specific primers binding to the *IghC* effector domain (Figure 1A).^27^

### DR maintains BCR repertoire diversity with age in the spleen

In line with previous work,^35^ we found that the splenic BCR repertoire was composed of IgM (∼61%), IgG (∼23%), IgA (∼13%), but few IgD and IgE (∼3%) (Figure S1G). The diversity of the BCR repertoire is defined by the number (richness) of clones and the relative frequency of the subdivisions of the clonal population. To assess whether DR affected BCR diversity, we calculated Hill diversity spectra.^36^^,^^37^ To determine whether DR mice had higher antigen-recognition capacity at old age, as reflected by evenness in the size of each B cell clone, we evaluated the Shannon diversity metric, a measure of both clonal richness and population structure, mostly affected by rare clones. We also calculated Simpson diversity, another measure of the distribution of the clonal population structure, mostly affected by large clones. AL mice displayed an age-related decline in both Shannon and Simpson indices (Figures 1C and 1D), mainly due to a decline in IgM, IgG, and IgE isotypes (Figures S2A and S2B). DR mice also showed an age-related decline in both diversity indices (Figures 1C and 1D), but this decline was less pronounced, leading to significantly higher Shannon and Simpson diversity in the spleen of DR animals at 24 months of age (Figures 1C and 1D; Table S1). DR mice exhibited significantly increased Shannon and Simpson diversity with age in IgE, and there was also a clear trend for DR to attenuate age-related changes in IgG, and at old age also in IgM (Figures S2A and S2B). Overall, the aging BCR repertoire showed loss of rare clones with age, indicated by the changes in the Shannon diversity (Figure 1C), and DR enhanced overall diversity by maintaining a more uniform distribution of large clones (Figure 1D).

To address whether switching to DR in mid-life is sufficient to recapitulate the beneficial effects of lifelong DR on the BCR repertoire at old age, we assessed AL_DR16M animals at 20 and 24 months of age (Figure 1B). Shannon diversity significantly increased in AL_DR16M mice compared to AL controls at 24 months, to levels indistinguishable from those in DR mice (Figure 1C; Table S1), suggesting a complete effect of later-onset DR. The AL_DR16M group showed a similar age-related loss in Shannon diversity with age to the DR mice (Figure 1C, Table S1). Similarly, there was a very strong trend of significantly increased Simpson in AL_DR16M compared to AL mice at 24 months, but the levels were not different compared to AL or DR mice at 20 and 24 months of age (Figure 1D; Table S1), indicating a partial effect of later-onset DR. IgD Shannon diversity was significantly higher in AL_DR16M compared to DR mice at 20 months (Figure S2A). In addition, IgM and IgE Shannon and Simpson diversity and IgA Simpson diversity were elevated in AL_DR16M relative to AL at 24 months of age, indicating a primary immune response (Figures S2A and S2B). Therefore, mid-life onset of DR at least partially converged with DR in BCR within-individual diversity between 20 and 24 months of age, with some of the mice showing an enhanced primary (IgM) and hypersensitivity (IgE) immune response to the switch to DR.

### DR and mid-life DR attenuate clonal expansions and inter-individual dissimilarity with age in the spleen

To determine whether the age-dependent decrease in within-individual antibody diversity was due to a B cell population skewed toward clonally expanded cells, we calculated clonal expansion as the percentage of the BCR repertoire taken up by the 20 most common clones (clonal abundance, P20) (Figure 1E). Clonal expansions increased progressively with age in AL mice (Figure 1E), in line with previous work.^7^^,^^15^ Clonal expansion was most evident in the primary and long-term antigen response isotypes IgM and IgG (Figure S2C), suggesting a possible attenuation of memory immune response.^28^ Clonal expansions also increased with age in DR mice (Figure 1E), but to a significantly lesser extent than in AL animals (Figure 1E). At 24 months of age, only ∼60% of the total clonal population was occupied by expanded clones in DR mice, while in AL mice it reached ∼80% (Figure 1E). Furthermore, mice under DR maintained a stable rate of clonal expansions in IgM and IgG, only increasing at 24 months of age in IgM (Figure S2C). The age-dependent decrease in within-individual diversity was thus associated with an age-related increase toward clonally expanded B cells, and this increase was attenuated by DR.

DR onset at 16 months also reduced the BCR repertoire clonal expansions. There was lower clonal expansion in AL_DR16M compared to AL at 24 months of age (Figure 1E) but no significant difference compared to DR (Figure 1E). Thus, onset of DR at 16 months of age was sufficient to reduce clonal expansion to the same extent as the lifelong DR treatment.

Differences in clonal composition of B cells between individuals are accentuated by the proliferation of different clones in different individuals.^7^^,^^13^^,^^15^^,^^38^ Expanded clones overrepresent a set of sequences that will be different by default between individuals having expansions of different clones. We therefore assessed inter-individual dissimilarity at different ages. To quantify dissimilarity, we used the repertoire dissimilarity index (RDI).^39^ The RDI is calculated in a five-step process including repertoire subsampling, counting the abundance of each feature (i.e., *IghV*, *IghD*, and *IghJ* gene segments) (Figures S1A and S1B), normalization of counts, pairwise comparisons between BCR repertoires, and calculating euclidean distances for each pair, and repeating this process 100 times to obtain an average RDI. Consistent with previous reports in mice and humans,^7^^,^^13^^,^^15^ RDI progressively increased with age in AL mice (Figure 1F), and in DR animals, but to a significantly lesser extent (Figure 1F). Isotype-specific analysis revealed that RDI increased with age in all isotypes in AL mice and all except IgA in DR mice (Figure S2D). However, the slope of the RDI was significantly reduced under DR in all isotypes except IgE (Figure S2D). Therefore, DR mitigated the progressive increase of inter-individual dissimilarity with age, likely attributable to a B cell repertoire that is less prone to clonal expansions and, thus, less heterogeneous between individuals.

We next evaluated whether mid-life-onset DR would also be sufficient to reduce the RDI. AL_DR16M mice had a significantly lower overall RDI than AL mice (Figure 1F). This reduction of RDI in DR animals was also significant 4 months after the start of DR, at 20 months, and at 24 months of age, similar to DR animals (Figure 1F; Table S1). Moreover, AL_DR16M mice experienced a reduction in IgE RDI when compared to AL and DR at 20 months of age (Figure S2D), and this stabilized to levels similar to AL and DR 4 months later. At 24 months, RDI of IgA and IgM was lower in AL_DR16M than in AL and DR (Figure S2D). Thus, mid-life DR also attenuated the age-related increase in inter-individual dissimilarity, consistent with the spike in diversity of IgE, IgA, and IgM.

### DR does not affect somatic hypermutation frequency, CDR3 length, or class-switch recombination in the spleen

Clonal diversity is important for efficient antigen recognition.^8^ We thus further studied whether antigen-recognition capacity is affected by aging and DR. We therefore evaluated the somatic hypermutation (SHM) frequency, which is the mechanism for affinity maturation of the BCR repertoire in response to antigen exposure, leading to clonal diversity.^8^^,^^28^ The frequency of synonymous substitutions, which do not result in a substitution of amino acids, indicates neutral evolution, providing a baseline for the non-synonymous substitutions, which do alter amino acids and accumulate during affinity maturation and become fixed under positive selection.^40^ Consistent with previous work,^8^ we did not detect any differences in synonymous or non-synonymous SHM frequency with age or diet (Figures S2E, S2F, and S3A–S3C).

Increased CDR3 length and variability has been causally associated with autoimmune disorders and old age.^10^^,^^41^ However, we found no changes in CDR3 length or variability with age in AL or DR mice (Figures S1E and S2G). Similarly, the analysis of BCR clone composition reported a significant loss in the pool of BCR clones where no naive isotypes remain (IgM^−^IgD^−^, post-antigenic) in AL mice with age (Figure S3D). DR mice displayed a non-significant trend for less IgM^+^IgD^+^SHM^−^ (naive) and more IgM^−^IgD^−^ clones at 20 and 24 months of age (Figure S3D). In line with these findings, there was a similar but weaker trend for the AL_DR16M (Figure S3D). These results suggest that aging may impair the class-switch recombination capacity.

### DR increases somatic hypermutation frequencies in the aged ileum

DR modulates the composition of the gut microbiome in mice and humans.^42^^,^^43^ As mucosal B cells in the gut are in direct contact with the gut microbiome,^44^^,^^45^^,^^46^^,^^47^ we examined the gut mucosal BCR repertoire. Within the small intestine, the highest accumulation of B cells is found in the Peyer’s patches in the ileum.^48^ Thus, we measured BCR repertoire dynamics in the ileum dependent on age and DR. Consistent with previous studies,^45^^,^^46^^,^^47^ IgA was the predominant isotype in the ileum, accounting for up to 98% of all Ig isotypes in young animals (Figure S3E). Again, in line with previous reports,^47^ with age there was an increase in IgM and IgG isotypes at the expense of IgA, which could be important for maintaining homeostasis with the intestinal microbiome (Figure S3E). No differences from AL in isotype abundances were detected in the ileum of DR or AL_DR16M animals (Figure S3E). Thus, DR did not affect the isotype structure of the intestinal antibody repertoire.

**Figure 2:**
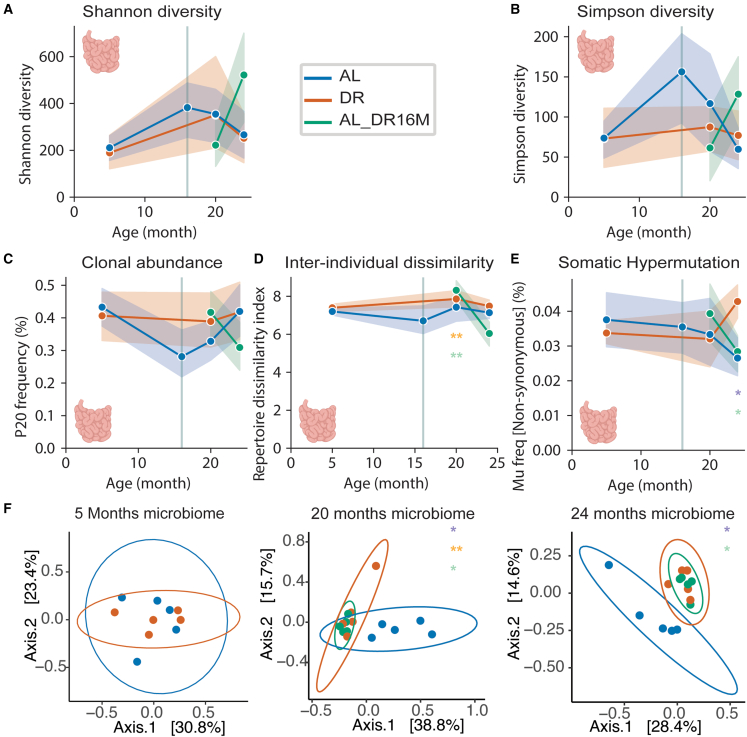
DR and aging only induce minor changes in the intestinal BCR repertoire (A) Shannon within-individual diversity. Significant differences through age and diet (two-way ANOVA): AL vs. AL_DR16 age × diet p = 0.019056; DR vs. AL_DR16 age × diet p = 0.042762. (B) Simpson within-individual diversity. Significant differences through age and diet (two-way ANOVA): AL vs. AL_DR16 age × diet p = 0.033592. (C) Clonal abundance. Significant differences through age and diet (two-way ANOVA): AL vs. AL_DR16M age × diet p = 0.03. (D) Inter-individual dissimilarity. Significant differences through age and diet (two-way ANOVA): AL vs. AL_DR16 age p = 0.000069; age × diet p = 0.00132; DR vs. AL_DR16 age p = 0.000011; age × diet p = 0.000809. Significant differences at 20 months of age (Mann-Whitney U test): AL_DR16M vs. AL p = 0.04; AL_DR16M vs. DR p = 0.009. (E) Frequency of non-synonymous SHM. Significant differences through age and diet (two-way ANOVA): DR vs. AL_DR16 age × diet p = 0.041. Significant differences at 24 months of age (Mann-Whitney U test): DR vs. AL p = 0.024; AL_DR16M vs. DR p = 0.024. (F) Microbiome unweighted UniFraq principal coordinates analysis and microbiome inter-individual dissimilarity at 5, 20, and 24 months of age. Ellipse represents 95% confidence interval. Significant differences at 20 months of age (PERMANOVA) DR vs. AL p = 0.011; AL_DR16M vs. DR p = 0.01; AL_DR16M vs. AL p = 0.011. Significant differences at 24 months of age (PERMANOVA) DR vs. AL p = 0.015; AL_DR16M vs. AL p = 0.012. (A–E) Lines correspond to mean, and shaded area to 95% confidence intervals. n = 5 female mice per treatment. ^∗^p < 0.05, ^∗∗^p <0.01 (Mann-Whitney U test) at individual time points represented in light purple for DR vs. AL, light green for AL vs. AL_DR16M, and orange for DR vs. AL_DR16M. For non-significant p values, see Table S1.

DR had only minor effects on the BCR within-individual diversity in the ileum. We detected no changes in diversity, clonal abundance, or inter-individual dissimilarity with age or with diet (Figures 2A–2D and S4A–S4D).

Previous studies have shown an age-associated decline in B cell selection processes in the gut, paired with declining SHM rate.^8^^,^^49^ We therefore evaluated whether DR influenced class-switch recombination and SHM. As previously reported in humans,^50^ the young gut BCR consisted primarily of IgM^−^IgD^−^ clones (∼73%), some IgM^+^IgD^+^SHM^+^ (antigen stimulated) clones (∼25%), and very few IgM^+^IgD^+^SHM^−^ clones (∼2%) (Figure S5A). There were no differences in the proportions of BCR clones in each class-switch recombination stage with age or under DR (Figure S5A). Consistent with previous work,^45^ DR treatment was characterized by positive selection of B cells through age (Figures S5B and S5C). Additionally, DR mice had significantly higher non-synonymous SHM compared to AL at 24 months of age (Figure 2E). Further, we found an age-associated decline in synonymous and non-synonymous SHM in IgM (Figures S5D and S5E). In addition, synonymous IgA SHM increased with age in DR mice (Figure S5D) and IgA non-synonymous SHM declined with age in AL (Figure S5E). Therefore, in the ileum, DR was associated with intact B cell selection and elevated SHM in old age.

To examine whether DR initiation at 16 months of age recaptured the SHM frequency preservation under DR treatment, we evaluated SHM frequencies in AL_DR16M mice. Surprisingly, non-synonymous SHM frequencies in 24-month-old mice were significantly lower in AL_DR16M and AL compared to DR (Figure 2E). This was consistent with SHM findings in IgA (Figure S5E), which might imply that onset of DR in mid-life does not affect affinity maturation.

Finally, CDR3 length and variability in the ileum were not affected by age or DR treatment (Figures S1F and S5F). Taken together, DR increases SHM of the ileal BCR repertoire by means of IgM and IgA isotypes.

### The aging microbiome responds to mid-life DR

The gut microbiome undergoes significant changes with age,^51^ primarily reflected in an age-dependent decline in within-individual diversity. This decline is accompanied by loss of beneficial bacteria and accumulation of commensal and pathogenic bacteria.^52^^,^^53^ DR positively affects the intestinal microbiome by maintaining a high abundance of bacteria considered to be beneficial for colonic health.^54^ Although B cells in the ileum are in direct contact with the gut microbiome,^44^^,^^45^^,^^46^^,^^47^ we only observed mild effects of DR and age on the ileum BCR repertoire, which might suggest that intestinal microbiome composition and BCR repertoire are mostly uncoupled. To directly address this hypothesis, we performed 16S rRNA amplicon sequencing^55^ on the V4 region of cecal pellets from AL and DR mice at 5, 20, and 24 months of age, and of AL_DR16M animals at 20 and 24 months of age. We found no difference in microbial within-individual diversity between diets (Figure S5G), consistent with a previous study in 28-month-old mice.^54^ In contrast, inter-individual dissimilarity in the microbiome, measured using the unweighted UniFraq diversity index, was significantly higher in AL when compared to DR at 20 and 24 months of age (Figure 2F). As opposed to the mild response of the gut BCR repertoire in AL_DR16M (Figures 2A–2E), the cecal microbiome of AL_DR16M diverged from the microbiome of AL mice already at 20 months of age, and this difference persisted at 24 months (Figure 2F). Therefore, inter-individual diversity rapidly responded to the switch from AL to DR, even when initiated at 16 months of age, indicating that the cecal microbiome is more susceptible to the change in diet than the ileum BCR repertoire.

### DR-related BCR metrics are correlated with healthier phenotypes

**Figure 3:**
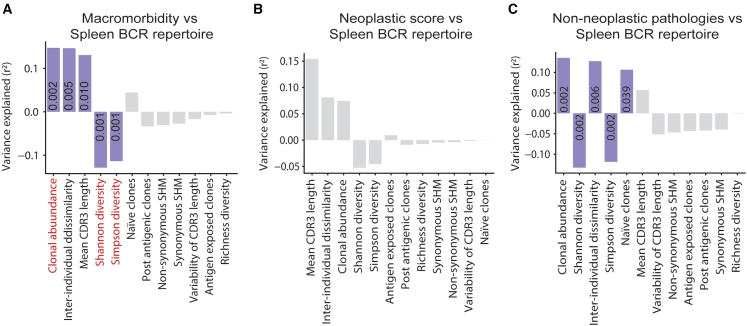
DR-associated BCR metrics are correlated with healthier phenotypes (Correlation of BCR metrics in the spleen with (A) macromorbidity, (B) neoplastic score, and (C) non-neoplastic pathologies burden. Colored bars indicate significant correlation (Spearman correlation p < 0.05). Red metrics are Bonferroni corrected p < 0.05. n = 5 female mice per treatment.

Finally, to characterize the observed BCR repertoire dynamic patterns and understand how they are reflected in host health, we tested the correlation between morbidity and systemic BCR metrics in the spleen (Figures 3A–3C). The “macromorbidity index” developed in the present work was adapted from various sources^56^^,^^57^^,^^58^ to encompass the collected macro-pathology of these mice. For each mouse, the macromorbidity index was calculated as the sum of neoplasia grade and non-neoplastic pathologies burden (Figures 3A–3C).

Of all the metrics obtained from the spleen BCR repertoire, clonal expansion, inter-individual dissimilarity, and mean CDR3 length were positively correlated with macromorbidity. Shannon and Simpson diversity metrics displayed a negative correlation (Figure 3A), indicating that higher within-individual diversity is correlated with a healthier state. Further, we asked which isotypes showed strongest correlation with morbidity and found that IgM and IgG underpin the correlation between morbidity and BCR characteristics (Figures S6A–S6C).

In addition, to determine whether the correlation of the BCR features with morbidity is attributed to neoplasia or non-neoplastic pathologies, the correlation was analyzed independently for neoplastic and non-neoplastic pathologies (Figures 3B and 3C). We found a significant correlation of clonal expansion, inter-individual dissimilarity, and Shannon and Simpson diversity with non-neoplastic pathologies in the spleen (Figure 3C), whereas no significant correlation of BCR features with neoplastic pathologies was detected (Figure 3B). Nevertheless, the variance explained by mean CDR3 length for neoplasia in the spleen, albeit not significant, was higher than the variance explained by clonal expansion, inter-individual dissimilarity, and Shannon and Simpson diversity for non-neoplastic pathologies (Figures 3B and 3C). Furthermore, the combined macromorbidity index yielded the strongest significant correlations of BCR features and morbidity (Figure 3A), suggesting that the individual analysis for neoplastic and non-neoplastic pathologies was underpowered. Therefore, both neoplasia and non-neoplastic pathologies contributed to the correlation of the BCR features with morbidity. Importantly, having found lower clonal expansion and RDI in the spleen of DR mice, paired with increased Shannon and Simpson diversity (Figures 1C–1G), our findings suggest that DR might delay the systemic functional decline of the BCR repertoire with age and be associated with a younger and healthier BCR repertoire. Similarly, decreased clonal expansion and RDI, and increased Shannon diversity in the spleen of AL_DR16M mice, might suggest that initiating DR later in life could recapitulate the profile of the DR BCR repertoire and be correlated with lower macromorbidity.

## Discussion

The profound impairment of immune function with aging is well documented,^4^^,^^5^^,^^6^^,^^59^ and strategies that can ameliorate age-associated immune dysregulation hold great promise to delay aging. DR, one of the most powerful anti-aging interventions known to date, affects immune homeostasis.^2^^,^^22^^,^^23^ In this study, we sequenced the BCR repertoire of spleen and ileum. We identified remodeling of BCR features in DR and AL_DR16M mice. We found low within-individual diversity, high clonal expansions, and high inter-individual dissimilarity correlated with morbidity, reflecting non-neoplastic pathologies. Given the direct link between the immune system and neoplasia,^60^ we expected that neoplasia would be a main contributor to the correlation of splenic BCR metrics with morbidity. However, we only obtained significant correlations of BCR metrics for non-neoplastic pathologies, which could be attributed to the limited statistical power of analyzing neoplasia and non-neoplastic pathologies separately. This is a plausible explanation given that the correlation of the combined macromorbidity index with BCR metrics was characterized by lower p values as compared to the correlation of non-neoplastic pathologies only to BCR metrics. Furthermore, classification of neoplasia in only three categories encompasses limited information on neoplastic pathologies and thus might also contribute to the absence of significant findings in the association of BCR metrics with neoplastic pathologies. Therefore, our findings suggest that both neoplastic and non-neoplastic pathologies are correlated with BCR metrics and highlight distinct patterns of the correlation of individual BCR metrics to each type of pathology; while mean CDR3 length seems to be more strongly correlated with neoplastic pathologies, clonal expansion, inter-individual dissimilarity, and Shannon and Simpson diversity show a stronger correlation to non-neoplastic pathologies. Our findings also suggest that the amelioration of the aging splenic BCR dysregulation by DR indicates that DR maintains a healthier, younger-like BCR repertoire. However, a limitation of the current study is the lack of information on the B cell subsets (e.g., marginal, follicular, B-1) encompassing the sequenced cell pool. Future studies performing fluorescence-activated cell sorting should not only investigate the B cell types involved in the DR response but also causally address whether DR improves vaccination outcomes due to “enrichment” of the BCR repertoire.

Our results highlight the importance of global splenic BCR repertoire dynamic metrics, such as within-individual variability, clonal expansions, and inter-individual dissimilarity in enhanced health under DR. Noteworthy, DR mice had an increased within-individual variability (Shannon diversity) in their BCR already at 5 months of age. This finding suggests not only that DR prevents age-related changes but that some effects are acute and already manifest in young animals. Consistently, DR started at 2 or 3 months of age has been shown to cause acute effects on T cell subsets, especially on naive T cells, in 6-month-old mice.^61^^,^^62^ Moreover, we also uncover intriguing aging- and DR-associated changes in Ig isotypes, as well as temporal changes that have been thus far unexplored in the context of anti-aging interventions. DR at 20 months of age was associated with increased splenic IgA compared to 5 months. In contrast, at 24 months, DR mice showed a strong increase in IgG abundance, while IgA was reduced. While it is currently unclear what causes this change, it might reflect age-related alterations in inflammation or dysregulated immune response, which happen mainly after 21–24 months of age.^63^ IgG has been shown to undergo altered glycosylation in response to obesity and disease-induced inflammatory states and with increased age.^64^^,^^65^^,^^66^^,^^67^ Glycosylation is a result of somatic hypermutation during antigen exposure driving specific IgG immune responses and leads to alterations in IgG effector functions and thereby represents one of the functional changes involved in pathological conditions. Interestingly, an increase in the levels of pro-inflammatory IgG glycans has been documented with aging.^68^ Therefore, the increased IgG abundance we observed in the spleen of 24-month-old DR mice could be indicative of a targeted immune response to counteract increased inflammatory levels at 24 months. Moreover, we found that the within-individual diversity of both IgM and IgG isotypes was correlated with macromorbidity, suggesting that preservation of high IgM and IgG diversity is one of the features of a healthier BCR repertoire. DR may offer a tighter regulation of the primary immune response, and thereby the maintenance of IgM clonal expansion rate might be a contributor to DR-mediated beneficial effects. Nevertheless, our study strictly reflects repertoire trajectories in non-pathogen-exposure conditions. Future studies where BCR isotypes are evaluated under more physiological conditions are necessary to more comprehensively reveal the implications of these changes on adaptive immune function and DR-associated beneficial effects.

We show that mid-life onset of DR is sufficient to improve many BCR metrics; e.g., increased Shannon diversity and attenuation of the increase in clonal expansions under DR were fully recapitulated by the mid-life diet switch. This poses the question of whether late-onset DR acutely rejuvenated these BCR metrics or just slowed them down after it was introduced. One limitation is that we lack a measurement soon after the DR switch and only measured the BCR repertoire 4 months post switch. Thus, it is not trivial to distinguish whether potential changes we see are the result of an acute reversal or a slow-down after the switch, or a combination of both. While there is some evidence that DR might rejuvenate specific parameters, e.g., in the spleen, Shannon diversity in IgG of AL_DR16M animals at 20 months of age was higher than the AL baseline at 16 months of age, which cannot simply be explained by delayed aging after the diet switch, but these differences were not statistically significant and therefore do not allow a clear conclusion. Therefore, future studies with higher time resolution and a higher number of biological replicates should be performed to answer the question of whether late-onset DR can rejuvenate the BCR repertoire.

We uncovered specific changes under AL_DR16M, including a spike in IgM, that could be relevant in understanding the implications of BCR repertoire responsiveness to a mid-life onset of DR. A previous study reported that onset of DR at 15 months of age improved hematopoietic regeneration of aging hematopoietic stem cells (HSCs).^69^ Therefore, the IgM spike under AL_DR16M could be indicative of a greater HSC regeneration capacity and facilitate the repertoire responsiveness in response to the dietary switch. Noteworthy, we found that AL_DR16M mice also respond to DR initiation through an increase in IgE diversity in spleen. Although elevated IgE levels are primarily implicated in allergic reactions,^28^^,^^70^^,^^71^ it is highly unlikely that food or other allergens contribute to the increased IgE diversity levels after DR onset at 16 months, given the clean housing conditions and the unchanged chow food composition. High IgE levels have been previously documented in germ-free or antibiotic-treated mice, typically characterized by low microbiome diversity, suggesting a regulatory role of the microbiome in controlling systemic IgE levels.^72^ Therefore, the spike in IgE in the AL_DR16M mice might reflect an acute loss of microbial within-individual diversity after DR onset. Nonetheless, future studies are necessary to confirm and comprehensively assess the changes and potential contributors in IgE responses in the context of dietary switches.

We show that the BCR repertoire of the ileum was not strongly affected by age or DR. In contrast, the cecal microbiome was significantly affected by DR and also acutely reacted to the late-life diet switch, suggesting that the BCR repertoire in the ileum is at least in part uncoupled from the microbiome. Consistently, microbiome transfer in the killifish did not cause changes in the ileum B cell compartment,^45^^,^^73^ further indicating that changes in the microbiome do not necessarily translate to changes in the ileum BCR repertoire. Even though there were overall only few changes in the ileum, DR and AL_DR16M altered some dynamics in isotype abundances. At 20 months of age, DR animals showed a strong trend of increased IgM and IgG abundance at the expense of IgA compared to 5-month-old animals. In contrast, at 24 months of age, IgM and IgG isotype abundances declined and IgA increased. Since inflammatory and immunosenescence signals dramatically increase in the gut after 21 months of age,^63^ elevated IgA levels between 20 and 24 months might be required to maintain homeostasis within the intestinal microbiome. After the diet switch, AL_DR16M mice showed an initial reduction in IgM and IgG abundance at 20 months of age, while, at 24 months, IgM and IgG abundance was increased. It was previously shown that IgA levels are reduced in the intestine of mice under high-fat diet.^74^ Thus, the trend of an initial reduction in IgM and IgG could be due to an increase in IgA abundance in response to a switch from AL to DR at the expense of IgM and IgG. At 24 months of age, the AL_DR16M mice showed lower morbidity compared to AL, strongly correlating with features of IgM and IgG isotypes, potentially explaining the trend of increased IgM and IgG abundance compared to AL, as opposed to the low levels of IgM and IgG previously reported in senescence mouse models.^75^

The most robust change in the gut BCR repertoire was that a declining aging SHM capacity was observed in AL mice, affecting predominantly the IgA isotype. Lindner et al. showed that neither antibiotic treatment nor diet modulate the IgA clonal composition in human colon or mouse small intestine.^45^ They postulated that, to maintain homeostasis through the interaction of the host and its microbiome, the IgA repertoire undergoes diversification of existing memory B cells instead of generating new B cell clones.^45^ This could be a result of the layer of early-life-origin B cells driving IgA responses in the adult gut,^76^ restricting the occurrence of drastic changes in the BCR repertoire of the ileum. Therefore, the decline in IgA SHM with age under AL feeding may be associated with impaired diversification and affinity maturation capacity, which might ultimately lead to disruption of host-microbiome balance and compromised mucosal defense.^45^^,^^77^ In contrast, DR may buffer the age-associated SHM decline observed in the AL mice ileum, indicating that DR feeding might offer an advantage in preserving diverse mucosal immune responses and gut homeostasis for extended periods.

In conclusion, in this study we show mitigation of the age-associated increase in clonal expansions with age and maintenance of within-individual diversity of the BCR repertoire in the spleen and ileum of mice under DR, correlating with improved mouse health, and we provide evidence that the splenic BCR repertoire responds to a later start of DR. Our findings also highlight the immune responsiveness of the mice where DR was initiated at 16 months of age, indicating that, even later in life, a short-term DR treatment can have beneficial effects on the adaptive immune system in mammals, a hypothesis that should be further explored in humans.

### Limitations of the study

As we only measured the BCR repertoire 4 months after the DR switch and did not include measurements shortly after the switch, we can currently not conclude whether DR rejuvenates the BCR repertoire or delays age-related changes after the switch. Further measurements with a higher time resolution after the diet switch will be necessary to address this question. Although BCR metrics were inversely correlated with morbidity under DR, this does not necessarily indicate a causal link between B cell repertoire features and morbidity but may be a consequence of reduced morbidity under DR. Furthermore, we did not examine the effects of DR on vaccination efficiency and the actual B cell subsets encompassing the sequenced cell pool. Future work performing vaccination studies and fluorescence-activated cell sorting are needed to functionally support our research findings and uncover the precise mechanisms by which DR affects the BCR repertoire.

## STAR★Methods

### Key resources table

**Table:** 

REAGENT or RESOURCE	SOURCE	IDENTIFIER
**Chemicals, peptides, and recombinant proteins**		

Trizol Reagent	Thermo Fisher Scientific	Cat#15596018
2x KAPA HiFi HotStart ReadyMix	KAPA Biosystems	Cat#07958935001
AMPure XP beads	Beckman Coulter	A63882

**Critical commercial assays**		

RNase-Free DNase Set	Qiagen	Cat#79254
Qubit® RNA BR Assay kit	Thermo Fisher Scientific	Cat#Q10211

**Deposited data**		

B cell receptor repertoire sequencing of spleen and ileum	This paper	ENA: PRJEB57899 and ENA: PRJEB59978
16S sequencing of caecal microbiome	This paper	ENA: PRJEB58684
Code used for analysis	This paper	https://github.com/carolinamonzo/analysis_BCR_DR https://github.com/carolinamonzo/CM_16S_cross-sectionalF245

**Experimental models: Organisms/strains**		

Mouse: F1 hybrid wild type mice (C3B6F1)	Charles River Laboratories (strain codes 626 and 027),	https://www.criver.com/products-services/find-model/jax-c3hheouj-mice?region=23 https://www.criver.com/products-services/find-model/c57bl6-mouse?region=23

Oligonucleotides	Table S1	Turchaninova et al.^27^ and Caporaso et al.^27^^,^^55^

**Software and algorithms**		

Data processing	pRESTO v0.5.13	Vander Heiden et al.^78^
V(D)J gene annotation	IgBLAST v1.17	Ye et al.^79^
Reference mouse BCR sequences	IMGT v3.40	Giudicelli et al.^80^
Clone definition	SHazaM v1.1	Gupta et al.^81^
Novel allele identification	TIgGER v1.0	Gadala-Maria et al.^82^
Germline definition and annotation	Change-O v1.1	Gupta et al.^81^
Repertoire dissimilarity index calculation	RDI v1.0	Bolen et al.^39^
CDR3 lengths normal distribution fitting	Scipy v1.4.2	Virtanan et al.^83^
Statistical analysis	python v3.7.3	https://www.python.org/downloads/release/python-373/
Increase/decrease evaluation through age	Scipy v1.4.2	Virtanan et al.^83^
two-way ANOVA analysis	Statsmodels v0.11	Seabold et al.^84^
BCR metric and macromorbidity index correlation evaluation	Scipy	Virtanan et al.^83^
Plot generation	Seaborn v0.10.1	https://seaborn.pydata.org/whatsnew/v0.10.1.html

### Resource availability

#### Lead contact

Further information and requests for resources and reagents should be directed to and will be fulfilled by the lead contact, Sebastian Grönke (sebastian.groenke@age.mpg.de).

#### Materials availability

This study did not generate new unique reagents.

### Experimental model and subject details

Relevant subject metadata can be found in the ENA: PRJEB57899, ENA: PRJEB59978 and ENA: PRJEB58684, and the github repositories: https://github.com/carolinamonzo/analysis_BCR_DR and https://github.com/carolinamonzo/CM_16S_cross-sectionalF245.

Any additional information required to reanalyze the data reported in this paper is available from the lead contact upon request.

#### Mouse husbandry and DR treatment

The effect of DR on the mice BCR repertoire was studied on female F1 hybrid wild type mice (C3B6F1). Three treatment groups, including: AL, DR, and mice switched from AL to DR at 16 (AL_DR16M) months of age, respectively, were used for tissue collection (n = 40 mice). The DR treatment was started at 3 months of age to avoid developmental effects. Food consumption of the AL group was measured weekly, and DR animals received 60% of the food amount consumed by AL-fed animals. While AL animals had constant access to food, DR animals were fed once per day in the morning. All animals were checked daily for their well-being and any deaths. Animals were fed a commercially available rodent chow (ssniff R/M-H autoclavable, ssniff, Spezialdiaüten, Germany) and were provided with sterile-filtered water *ad libitum*. Chow was enriched with essential vitamins and minerals, ensuring that DR animals were adequately supplied with all required nutrients, despite their lower food intake. Mice were kept in the Comparative Biology facility at the Max Planck Institute for Biology of Aging. Animals were housed in groups of 5 females in individually ventilated cages (GM500 Mouse IVC Green Line, Tecniplast) under specific-pathogen-free conditions with constant temperature (21°C), 50–60% humidity and a 12-h light–dark cycle. For environmental enrichment, mice had constant access to nesting material and chew sticks. All protocols involving animals were carried out in accordance with the recommendations and guidelines of the Federation of the European Laboratory Animal Science Association, with all protocols approved by the Landesamt fuür Natur, Umwelt und Verbraucherschutz, Nordrhein-Westfalen, Germany (reference no. AZ: 84-02.04.2015.A437). Lifespan survival curves of AL and chronic DR animals were previously published.^85^

### Method details

#### Tissue collection and postmortem pathology/necropsy

Tissues were collected at 5, 16, 20 and 24 months of age. Therefore, mice were killed by cervical dislocation and tissues were snap frozen in liquid nitrogen and kept at −80°C. Each animal underwent cross-sectional pathology during tissue collection. Each dissected animal’s tumor load and other anomalies, including pathological appearance of organs (discoloration, enlargement, granular texture, etc.) were noted.

#### RNA isolation

Spleen and ileum samples were homogenised using a FastPrep-24 (MP Biomedicals) and the following program: 6 times bead-beating at 4 m/s for 30 s. RNA was isolated using 1 mL Trizol Reagent (Thermo Fisher Scientific, Germany) according to the manufacturer’s protocol before samples were treated with DNase using the RNase-Free DNase Set (Qiagen). Qubit RNA BR Assay kit (ThermoFisher Scientific) and Agilent TapeStation System (Agilent Technologies) were used to measure RNA quantity and quality.

#### Reverse transcription and library preparation for BCR-Sequencing

First strand cDNA synthesis with template switch and molecular barcoding, ultimately leading to BCR sequencing were performed according to^27^ with minor modifications. cDNA was synthesised from 600 ng of isolated RNA, purified and eluted in 10 μL nuclease-free water. A three-stage PCR amplification of the cDNA library was performed. First PCR added a second strand to the reverse-transcribed cDNA, and included nested primers binding to the (VDJ-) constant region that allow for Igh isotype identification. 1.5 μL of spleen or 5 μL of ileum cDNA were used for the first PCR reaction. 1μL of purified PCR1 product was used for the second PCR reaction. PCRs were done using the 2x KAPA HiFi HotStart ReadyMix (KAPA Biosystems) in a total volume of 25μL. DNA concentration and size distribution of libraries were determined using the Qubit dsDNA BR (Broad Range) Assay Kit (ThermoFisher Scientific) and TapeStation 4200 System (Agilent Technologies), respectively. Samples tagged with different internal barcodes were pooled in equal molar ratios in groups of 10 samples, yielding a total of 10 libraries, which were further purified. Purifications were performed using 0.8x volume of AMPure XP beads (Beckman Coulter). Illumina adaptors were ligated and asymmetric 400 + 100-nt paired-end sequencing was performed on an Illumina NovaSeq 600, at the sequencing core facility of the Max Planck Institute for Molecular Genetics in Berlin.

#### Caecal DNA extraction and library preparation for 16S-rRNA sequencing

Caecal content extracted directly from the gut of mice after cervical dislocation, frozen in liquid nitrogen and stored at −80°C, were used for 16S-rRNA sequencing. 5 to 40 mg of caecal content were homogenized by bead-beating with two 7 mm diameter stainless steel beads in a TissueLyzer II (Qiagen, 85300) for 30 s at 25 Herz/s. Next, 300 μl of DNA extraction buffer (27 mL SDS and 540 mg lysozyme shaked at 100 rpm for 20 min at 38°C) and 2 spoonfuls (43 mm) of zirconia beads (BioSpec) were added and bead-beated twice for 3 min at 30 Herz/s. After 6 min centrifugation at 4000 g (15°C), 80 μL were transferred to a 96 well plate. 2 μL RNAse A solution (Qiagen) were added, and plates were incubated for 30 min at 37°C. Another 1-h incubation at 56°C followed after the addition of 10 μL of Proteinase K (ThermoFisher) and 10 μL 20% SDS (ThermoFisher). 40 μL IRS solution (Qiagen) were added and plates were mixed and incubated for 5 min at 4°C. After spinning the plates for 5 min at 2000 g, 100 μL of the supernatant were transferred into a new 96 well plate. DNA was purified using the standard protocol for CleanNGS beads (GC biotech BV) at 1x concentration. Subsequently, purified DNA was quantified using PicoGreen assay (Lumiprobe), diluted to 5 ng/μL in nuclease-free water and amplified in two successive rounds of PCR, following the standard Illumina protocol. Briefly, the V4 region was amplified using primers 515F-806R^55^ to which dual-index barcodes of the Nextera XT kit were added. All PCR-product purifications were performed using 0.8x CleanNGS beads (GC biotech BV). For each of the obtained libraries, total concentration, as well as amplicon verification were determined using PicoGreen (Lumiprobe) and Agilent TapeStation 4200 System (Agilent Technologies). Samples were pooled in equal molar portions and 250bp paired-end sequencing was performed on an Illumina HiSeq 2500 (Admera Health).

### Quantification and statistical analysis

#### BCR data processing

Data processing was performed using the pRESTO (v0.5.13)^78^ tool from the Immcantantion framework. This included quality filtering (minQ = 20), demultiplexing by internal barcodes, primer masking, UMI extraction, generation of consensus reads from common UMIs, assembly of read pairs and annotation of IgH isotypes. A mean of 208089 UMI-consensus reads per sample were studied in the spleen, and 61724 in the ileum. We found no batch effect, due to either age or diet, on the number of UMI-consensus reads evaluated per mouse. Using IgBLAST (v1.17),^79^ V(D)J genes were annotated from reference mouse BCR sequences obtained from IMGT (v3.40).^80^ A data-table in AIRR (v1.3) standard format^86^ was built for analysis using the Change-O tool (v1.1).^81^ Un-productive sequences were filtered out and clones were defined using sample-specific thresholds calculated with the R (v4.0.3) package SHazaM (v1.1). Novel alleles were identified using TIgGER (v1.0),^82^ and germlines were defined and annotated with Change-O.

Quantification of clonal diversity, expansion and CDR3 region analysis were performed using Alakazam (v1.1). Clonal expansion was evaluated using the P20 metric, calculated as the sum of frequencies of all clones with rank above or equal to 20. SHM was quantified with SHazaM. Repertoire dissimilarity index for inter-individual dissimilarity was calculated using RDI (v1.0),^39^ and isotype frequency was calculated as percentage of the total reads corresponding to each isotype. Center and deviation of CDR3-length, Gaussian distributions and variability between biological replicates, were calculated to evaluate differences throughout age and between diet groups. CDR3 lengths were fitted on a normal distribution using Scipy (v1.4.2),^83^ and V-J gene usage was calculated using Alakazam.

#### Morbidity index

For each mouse, the macromorbidity index was calculated as the sum of the non-neoplastic pathologies burden, and the neoplasie grade. Neoplastic pathologies were identified in connective tissues, liver and lungs, while non-neoplastic pathologies included pathological appearance (bad habitus or kyphosis), enlarged spleen, discoloration of WAT (brown), knotty or granular pancreas, uterine cysts, enlarged kidneys and enlarged adrenal glands. Neoplasia were graded as 0 (absence of tumors), 1 (1 organ affected by tumors), or 2 (2 or more organs affected by tumors, representing metastatic cancer) and a degree of 1 was assigned to each non-neoplastic pathological finding at dissection.

#### Statistical analysis of BCR data

Statistical analysis was performed in python (v3.7.3). Linear regressions for AL and DR were calculated to evaluate increase/decrease through age (Scipy, v1.4.2),^83^ and two-way ANOVA (Statsmodels, v0.11)^84^ to compare the two diets. Mann-whitney U tests and Bonferroni multiple testing corrections were used to compare diets within each time point. The relationship between each BCR metric and the macromorbidity index was calculated using linear regression and spearman correlation (Scipy).^83^ All statistical details and outcomes of experiments can be found in the figure legends. Plots were generated using Seaborn (v0.10.1).

#### 16S-rRNA data processing and analysis

Raw-sequencing data was processed using cutadapt (v3.4)^87^ for quality filtering (minQ = 20), discarding reads with no primers, and removing full primer sequences. Dada2 (v1.18)^88^ was used for error correction, chimaera removal, assembly of read pairs and taxa annotation with the Silva database (v132).^89^ Bacterial phylogenetic trees were calculated using phyloseq (v1.34)^90^ for microbial within-individual diversity (Shannon) and inter-individual dissimilarity (Unweighted UniFraq). Statistical analyses were performed using vegan (v2.5.7) in R (v4.0.2), and were corrected for multiple testing using Benhamini Hochberg. All statistical details and outcomes of experiments can be found in the figure legends.

## Data Availability

• FASTQ files and mouse-specific metadata have been deposited to the European Nucleotide Archive (ENA) in the European Bioinformatics Institute (EMBL-EBI) (https://www.ebi.ac.uk/ena/browser/home) and are publicly available as of the date of publication. B cell receptor repertoire sequencing and meta-data of spleen and ileum were deposited in ENA: PRJEB57899 and ENA: PRJEB59978. 16S rRNA sequencing and meta-data of caecal microbiome were deposited in ENA: PRJEB58684. • All original code has been deposited on github (https://github.com/) and is publicly available as of the date of publication. Code for data processing and analysis of B cell receptor repertoire has been deposited at (https://github.com/carolinamonzo/analysis_BCR_DR), and code for data processing and analysis of 16S rRNA of caecal microbiome has been deposited at (https://github.com/carolinamonzo/CM_16S_cross-sectionalF245). • Any additional information required to reanalyze the data reported in this paper is available from the lead contact upon request.

## Supplemental information

Document S1. Figures S1–S6 and Table S2

Table S1. Supplementary statistics (A) Spleen relative abundance isotypes. (B) Spleen richness diversity. (C) Spleen Shannon diversity. (D) Spleen Simpson diversity. (E) Spleen clonal expansions. (F) Spleen RDI. (G) Spleen SHM synonymous. (H) Spleen SHM non-synonymous. (I) Spleen CDR3 length distribution. (J) Spleen class-switch status. (K) Ileum relative abundance isotypes. (L) Ileum richness diversity. (M) Ileum Shannon diversity. (N) Ileum Simpson diversity. (O) Ileum clonal expansions. (P) Ileum RDI. (Q) Ileum SHM synonymous. (R) Ileum SHM non-synonymous. (S) Ileum CDR3 length distribution. (T) Ileum class-switch status.

Document S2. Article plus supplemental information
