# Supplementary material for: Dietary restriction mitigates the age-associated decline in mouse B cell receptor repertoire diversity
Source: Cell Rep. 2023 Jun 28;42(7):112722. doi: 10.1016/j.celrep.2023.112722 (PMC10391628; doi:10.1016/j.celrep.2023.112722)
Supplement: Document S1. Figures S1–S6 and Table S2 [file mmc1.pdf]

Cell Reports, Volume *42*

Supplemental information

Dietary restriction mitigates the age-associated

decline in mouse

B cell receptor repertoire diversity

Carolina Monzó, Lisonia Gkioni, Andreas Beyer, Dario Riccardo Valenzano, Sebastian Grönke, and Linda Partridge

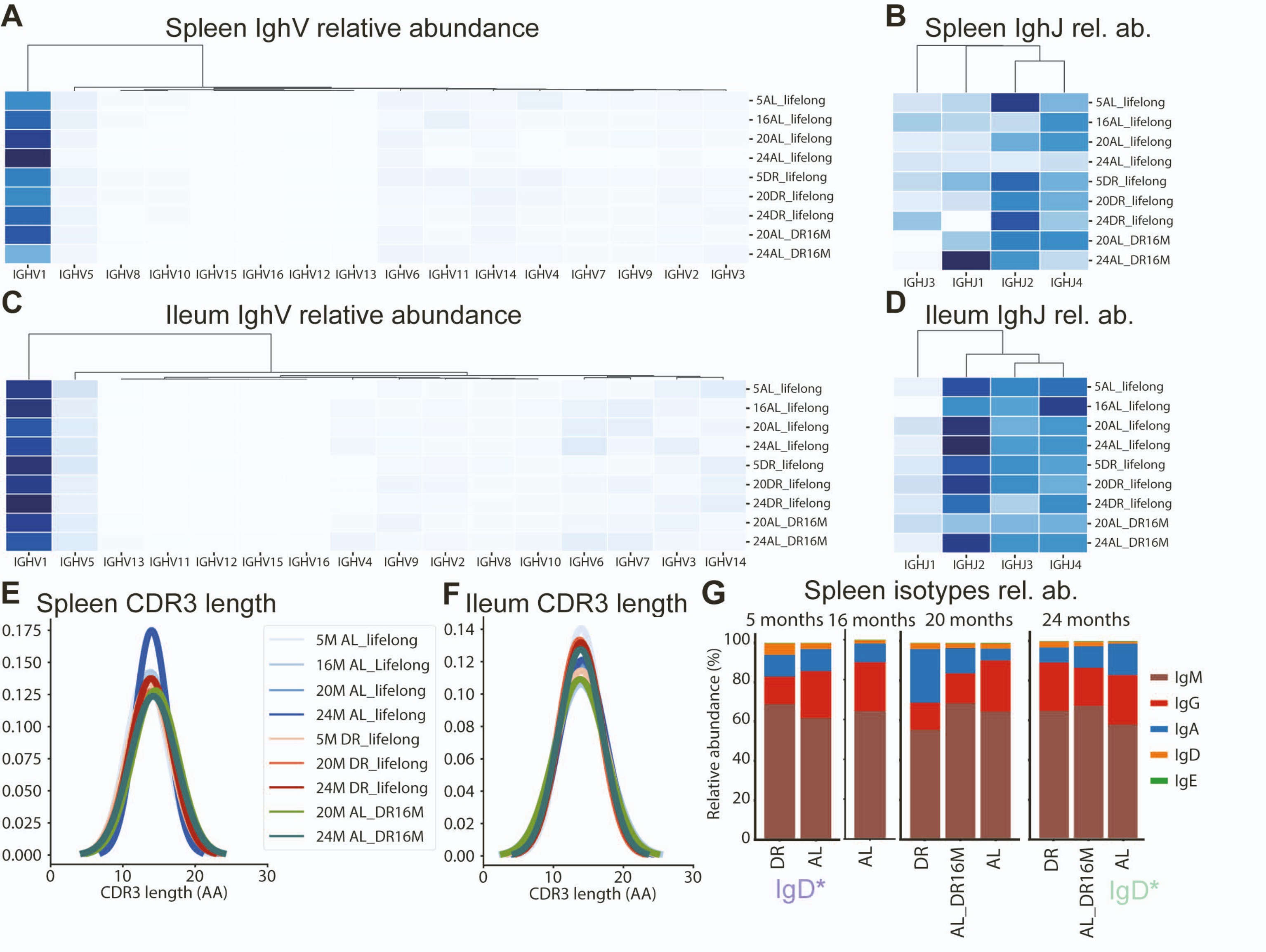
A
Spleen IghV relative abundance
5AL_lifelong
16AL_lifelong
20AL_lifelong
24AL_lifelong
5DR_lifelong
20DR_lifelong
24DR_lifelong
20AL_DR16M
24AL_DR16M
IGHV1 IGHV5 IGHV8 IGHV10 IGHV15 IGHV16 IGHV12 IGHV13 IGHV6 IGHV11 IGHV14 IGHV4 IGHV7 IGHV9 IGHV2 IGHV3
B
Spleen IghJ rel. ab.
IGHJ3 IGHJ1 IGHJ2 IGHJ4
C
Ileum IghV relative abundance
IGHV1 IGHV5 IGHV13 IGHV11 IGHV12 IGHV15 IGHV16 IGHV4 IGHV9 IGHV2 IGHV8 IGHV10 IGHV6 IGHV7 IGHV3 IGHV14
D
Ileum IghJ rel. ab.
IGHJ1 IGHJ2 IGHJ3 IGHJ4
E
Spleen CDR3 length
CDR3 length (AA)
5M AL_lifelong
16M AL_Lifelong
20M AL_lifelong
24M AL_lifelong
5M DR_lifelong
20M DR_lifelong
24M DR_lifelong
20M AL_DR16M
24M AL_DR16M
F
Ileum CDR3 length
CDR3 length (AA)
G
Spleen isotypes rel. ab.
5 months 16 months 20 months 24 months
Relative abundance (%)
IgM
IgG
IgA
IgD
IgE
DR AL AL DR AL_DR16M AL DR AL_DR16M AL
IgD*
IgD*

Figure S1. IghV/IghJ gene usage and CDR3 length in spleen and ileum. Related to Figure 1.

A) Mean relative *IghV* gene usage in the spleen BCR. Significant changes through age (linear regression): *IghV1* (AL p = 0.03), *IghV3* (AL p = $8.2x10^{-5}$), *IghV4* (AL p = 0.001; DR p = 0.001), *IghV6* (DR p = 0.02), IghV7 (AL p = 0.02; DR p = 0.02), and *IghV11 (AL p = 0.049; DR p = 0.03)*. Significant changes through age and diet (2-way ANOVA): *IghV3* (Age * Diet p = 0.01), *IghV15* (DR vs AL p = 0.02). **B)** Mean relative *IghJ* gene usage in the spleen BCR. **C)** Mean relative *IghV* gene usage in the ileum BCR. **D)** Mean relative *IghJ* gene usage in the ileum BCR. **E)** CDR3 length gaussian distribution in the spleen. **F)** CDR3 length gaussian distribution in the ileum. **G)** Relative abundance of antibody isotypes in the spleen. Significant differences through age (linear regression): IgD (AL p = 0.001; DR p = 0.002). Significant differences through age and diet (2-way ANOVA): IgD (AL vs DR Age p = 0.0001; AL vs DR Diet p = 0.01; AL vs AL_DR16M Age p = 0.03). Significant differences at 5 months of age (Mann-Whitney U test): IgD (DR vs AL p = 0.036). Significant differences at 24 months of age (Mann-Whitney U test): IgD (AL_DR16M vs AL p = 0.036). n= 5 female mice per treatment. *p-value < 0.05 at individual timepoints represented in light purple for DR vs AL and light green for AL vs AL_DR16M.

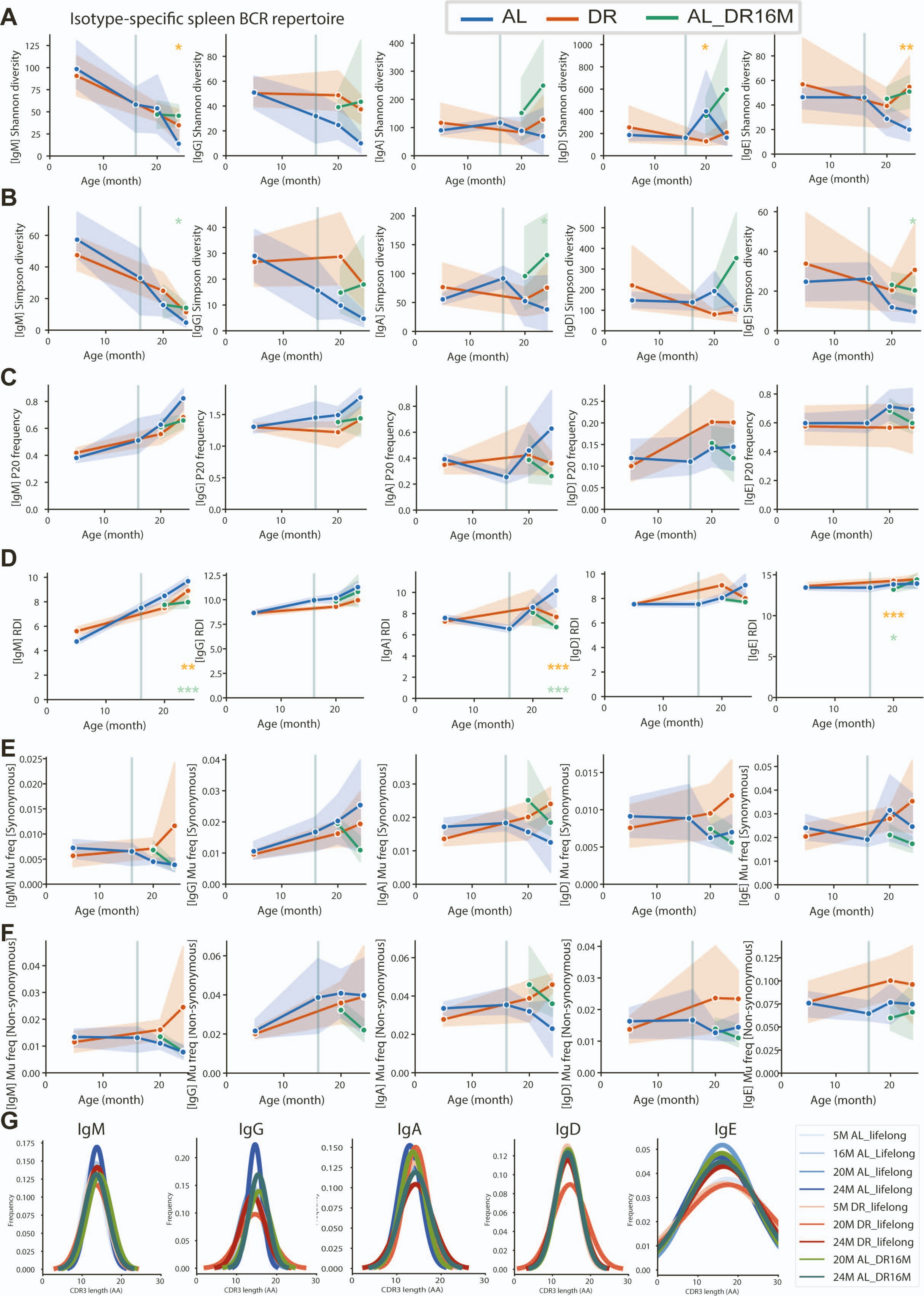

A
Isotype-specific spleen BCR repertoire
AL
DR
AL_DR16M
[IgM] Shannon diversity
[IgG] Shannon diversity
[IgA] Shannon diversity
[IgD] Shannon diversity
[IgE] Shannon diversity
Age (month)
B
[IgM] Simpson diversity
[IgG] Simpson diversity
[IgA] Simpson diversity
[IgD] Simpson diversity
[IgE] Simpson diversity
C
[IgM] P20 frequency
[IgG] P20 frequency
[IgA] P20 frequency
[IgD] P20 frequency
[IgE] P20 frequency
D
[IgM] RDI
[IgG] RDI
[IgA] RDI
[IgD] RDI
[IgE] RDI
E
[IgM] Mu freq [Synonymous]
[IgG] Mu freq [Synonymous]
[IgA] Mu freq [Synonymous]
[IgD] Mu freq [Synonymous]
[IgE] Mu freq [Synonymous]
F
[IgM] Mu freq [Non-synonymous]
[IgG] Mu freq [Non-synonymous]
[IgA] Mu freq [Non-synonymous]
[IgD] Mu freq [Non-synonymous]
[IgE] Mu freq [Non-synonymous]
G
IgM
IgG
IgA
IgD
IgE
Frequency
CDR3 length (AA)
5M AL_lifelong
16M AL_Lifelong
20M AL_lifelong
24M AL_lifelong
5M DR_lifelong
20M DR_lifelong
24M DR_lifelong
20M AL_DR16M
24M AL_DR16M

Figure S2. Isotype-specific BCR repertoire metrics of the spleen. Related to Figure 1. **A)** Shannon within-individual diversity. Significant differences through age (linear regression): IgM (AL p = 0.001; DR p = 0.001), IgG (AL p = 0.002), IgE (AL p = 0.01). Significant differences at 20 months of age (Mann-Whitney U test): IgD (AL_DR16M vs DR p = 0.02). Significant differences at 24 months of age (Mann-Whitney U test): IgM (AL_DR16M vs DR p = 0.04), IgE (AL_DR16M vs DR p = 0.002). **B)** Simpson within-individual diversity. Significant differences through age (linear regression): IgM (AL p = 0.0005; DR p = 0.0007), IgG (AL p = 0.004), IgE (AL p = 0.01). Significant differences at 24 months of age (Mann-Whitney U test): IgM (AL_DR16M vs AL p = 0.02), IgA (AL_DR16M vs AL p = 0.03), IgE (AL_DR16M vs AL p = 0.04). **C)** Clonal expansion. Significant differences through age (linear regression): IgM (AL p = 0.00012), IgG (AL p = 0.002). **D)** Inter-individual dissimilarity. Significant differences through age (linear regression): IgM (AL p = 8.2×10^{-21}; DR p = 1.1x10-16), IgG (AL p = 3.2×10^{-9}; DR p = 1.1x10-10), IgA (AL p = 0.004), IgD (AL p = 0.0022; DR p = 0.006), IgE (DR p = 0.005). Significant differences through age and diet (2-way ANOVA): IgM (Age * Diet p = 2.0x10-8), IgG (Age * Diet p = 0.00001), IgA (Age * Diet p = 0.0008), IgD (Age * Diet p = 0.000004). Significant differences at 20 months of age (Mann-Whitney U test): IgE (AL_DR16M vs AL p = 0.011; AL_DR16M vs DR p = 0.00005). Significant differences at 24 months of age (Mann-Whitney U test): IgM (AL_DR16M vs AL p = 1.0×10^{-6}; AL_DR16M vs DR p = 0.005), IgA (AL_DR16M vs AL p = 1.0×10^{-6}; AL_DR16M vs DR p = 0.0005). Significant differences between baseline AL at 16 months of age and mice at 20 months of age (Mann-Whitney U test): IgA (AL vs AL p = 0.01), IgD (AL vs AL p = 0.03; AL vs AL_DR16M p = 0.007), IgM (AL vs AL p = 0.015)**. E)** Frequency of synonymous SHM. Significant differences through age (linear regression): IgG (DR p = 0.038), IgA (DR p = 0.014). Significant differences through age and diet (2-way ANOVA): IgA (Age * Diet p = 0.01). **F)** Frequency of non-synonymous SHM. Significant differences through age and diet (2-way ANOVA): IgA (Age * Diet p = 0.009). **G)** CDR3-length distribution. **A-F)** Blue represents AL, red DR and green AL_DR16M. Lines correspond to mean, and shaded area to 95% confidence intervals. n= 5 female mice per treatment. *p-value < 0.05, **p-value < 0.01, ***p-value < 0.01 (Mann-Whitney U test) at individual timepoints represented in light green for AL vs AL_DR16M and orange for DR vs AL_DR16M.

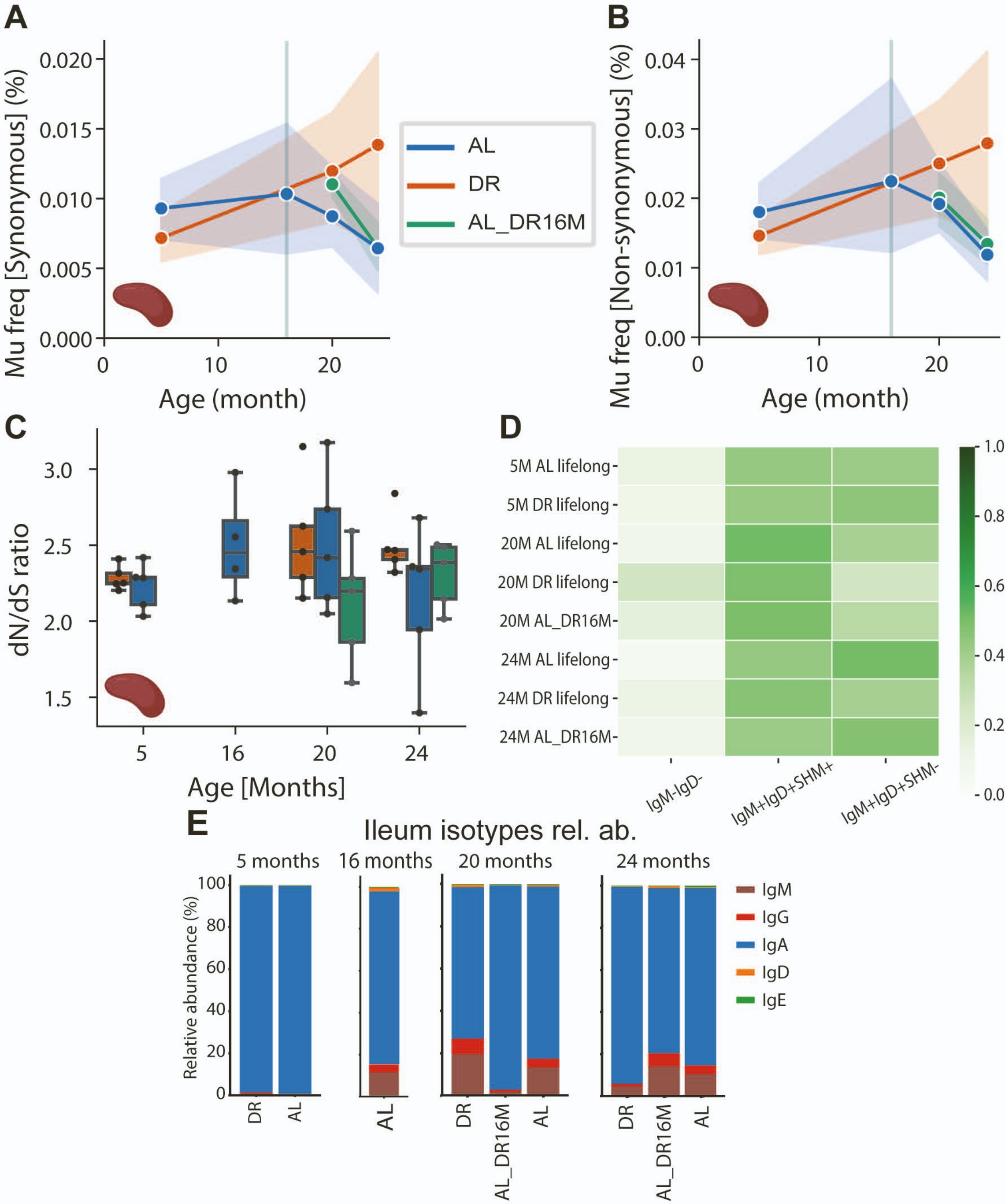

A
B
C
D
E
Mu freq [Synonymous] (%)
Mu freq [Non-synonymous] (%)
Age (month)
AL
DR
AL_DR16M
dN/dS ratio
Age [Months]
5M AL lifelong
5M DR lifelong
20M AL lifelong
20M DR lifelong
20M AL_DR16M
24M AL lifelong
24M DR lifelong
24M AL_DR16M
IgM-IgD-
IgM+IgD+SHM+
IgM+IgD+SHM-
Ileum isotypes rel. ab.
5 months
16 months
20 months
24 months
Relative abundance (%)
IgM
IgG
IgA
IgD
IgE
DR
AL
AL_DR16M

Figure S3. Somatic hypermutation frequency and relative abundance of antibody isotypes in the ileum. Related to Figure 2.

A) Frequency of synonymous SHM in the spleen. Significant differences through age and diet (2-way ANOVA): AL vs DR Age*Diet p = 0.0387; AL vs AL_DR16M Age p = 0.018. **B)** Frequency of non-synonymous SHM in the spleen. Significant differences through age and diet (2-way ANOVA): AL vs DR Age*Diet p = 0.035708; AL vs AL_DR16M Age p = 0.0104. Lines correspond to mean, and shaded area to 95% confidence intervals. **C)** Ratio of non-synonymous to synonymous mutations in spleen. Boxes indicate quartiles, and whiskers the rest of the data distribution. **D)** Relative frequency of clones according to their class-switch status in the spleen. Significant differences through age in AL (linear regression): p = 0.034. **E)** Relative abundance of antibody isotypes in the ileum. Significant differences through age (linear regression): IgG (AL p = 0.03). Significant differences between 5 and 20 months of age (Mann-Whitney U test): IgM (AL p = 0.036), IgG (AL p = 0.036), IgA (AL p = 0.024). n= 5 female mice per treatment.

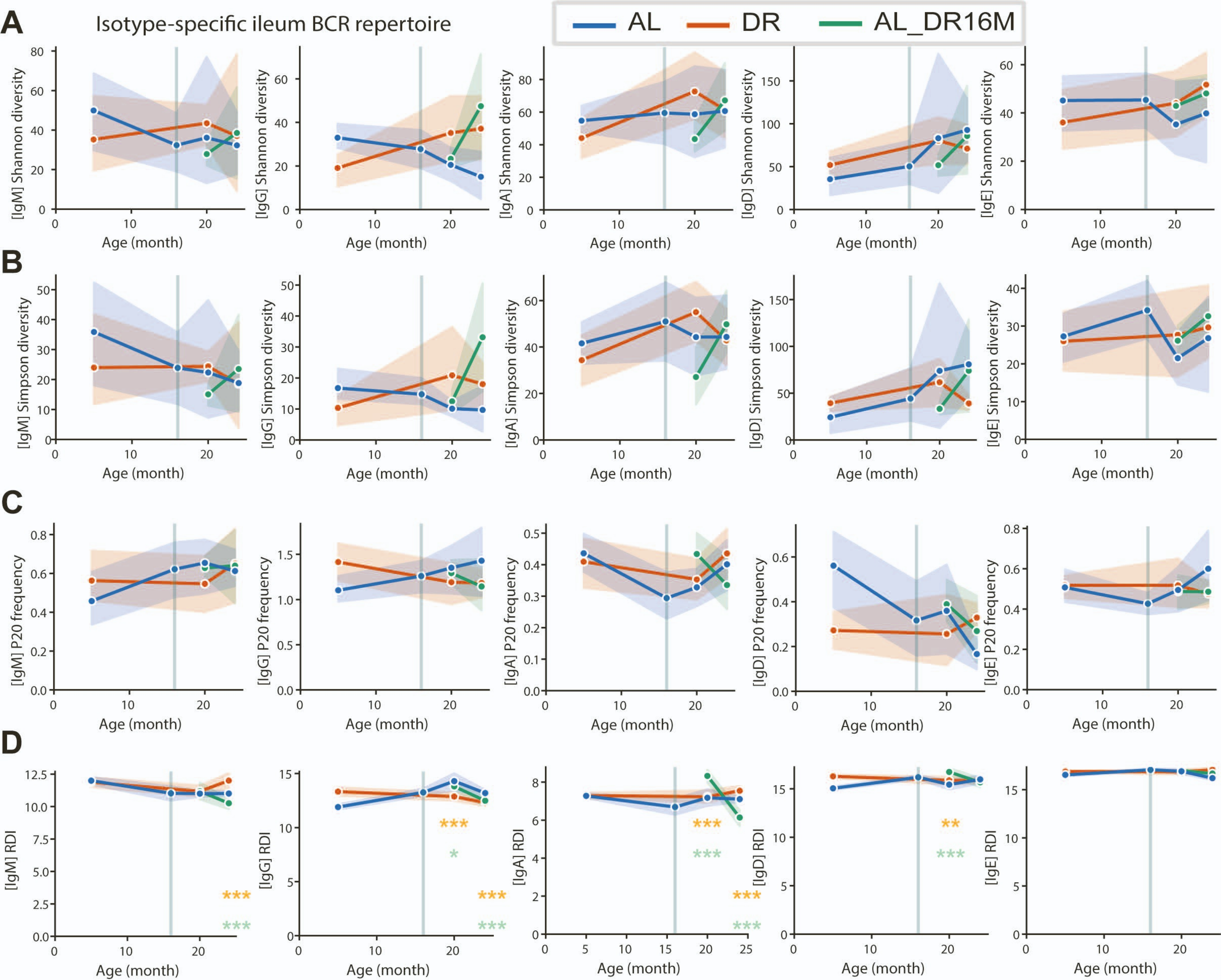

A
Isotype-specific ileum BCR repertoire
AL
DR
AL_DR16M
[IgM] Shannon diversity
[IgG] Shannon diversity
[IgA] Shannon diversity
[IgD] Shannon diversity
[IgE] Shannon diversity
Age (month)
B
[IgM] Simpson diversity
[IgG] Simpson diversity
[IgA] Simpson diversity
[IgD] Simpson diversity
[IgE] Simpson diversity
C
[IgM] P20 frequency
[IgG] P20 frequency
[IgA] P20 frequency
[IgD] P20 frequency
[IgE] P20 frequency
D
[IgM] RDI
[IgG] RDI
[IgA] RDI
[IgD] RDI
[IgE] RDI
***
*
**

Figure S4. Isotype-specific BCR repertoire metrics of the ileum. Related to Figure 2.
A) Shannon within-individual diversity. Significant differences through age (linear regression): IgD (AL $p = 0.03$), IgG (AL $p = 0.02$). Significant differences through age and diet (2-way ANOVA): IgG (Age * Diet $p = 0.006$). **B)** Simpson within-individual diversity. Significant differences through age (linear regression): IgD (AL $p = 0.025$). **C)** Clonal expansion. Significant differences through age (linear regression): IgG (AL $p = 0.003$). Significant differences through age and diet (2-way ANOVA): IgD (Age * Diet $p = 0.01$), IgG (Age * Diet $p = 0.042$)**. D)** Inter-individual dissimilarity. Significant differences through age (linear regression): IgM (AL $p = 4.0x10^{-5}$), IgG (AL $p = 4.0x10^{-6}$; DR $p = 2.9x10^{-6}$), IgD (AL $p = 0.003$; DR $p = 0.003$). Significant differences through age and diet (2-way ANOVA): IgM (Age * Diet $p = 0.00045$), IgG (Age * Diet $p = 9.4x10^{-19}$), IgD (Age * Diet $p = 0.000006$). *p-value < 0.05, **p-value < 0.01, ***p-value < 0.01 (Mann-Whitney U test) at individual timepoints represented in light green for AL vs AL_DR16M and orange for DR vs AL_DR16M. Significant differences between baseline AL at 16 months of age and mice at 20 months of age (Mann-Whitney U test): IgG (AL vs AL $p = 0.036$), IgD (AL vs AL_DR16M $p = 0.036$), IgA (AL vs AL_DR16M $p = 6.0x10^{-6}$). Lines correspond to mean, and shaded area to 95% confidence intervals. n= 5 female mice per treatment.

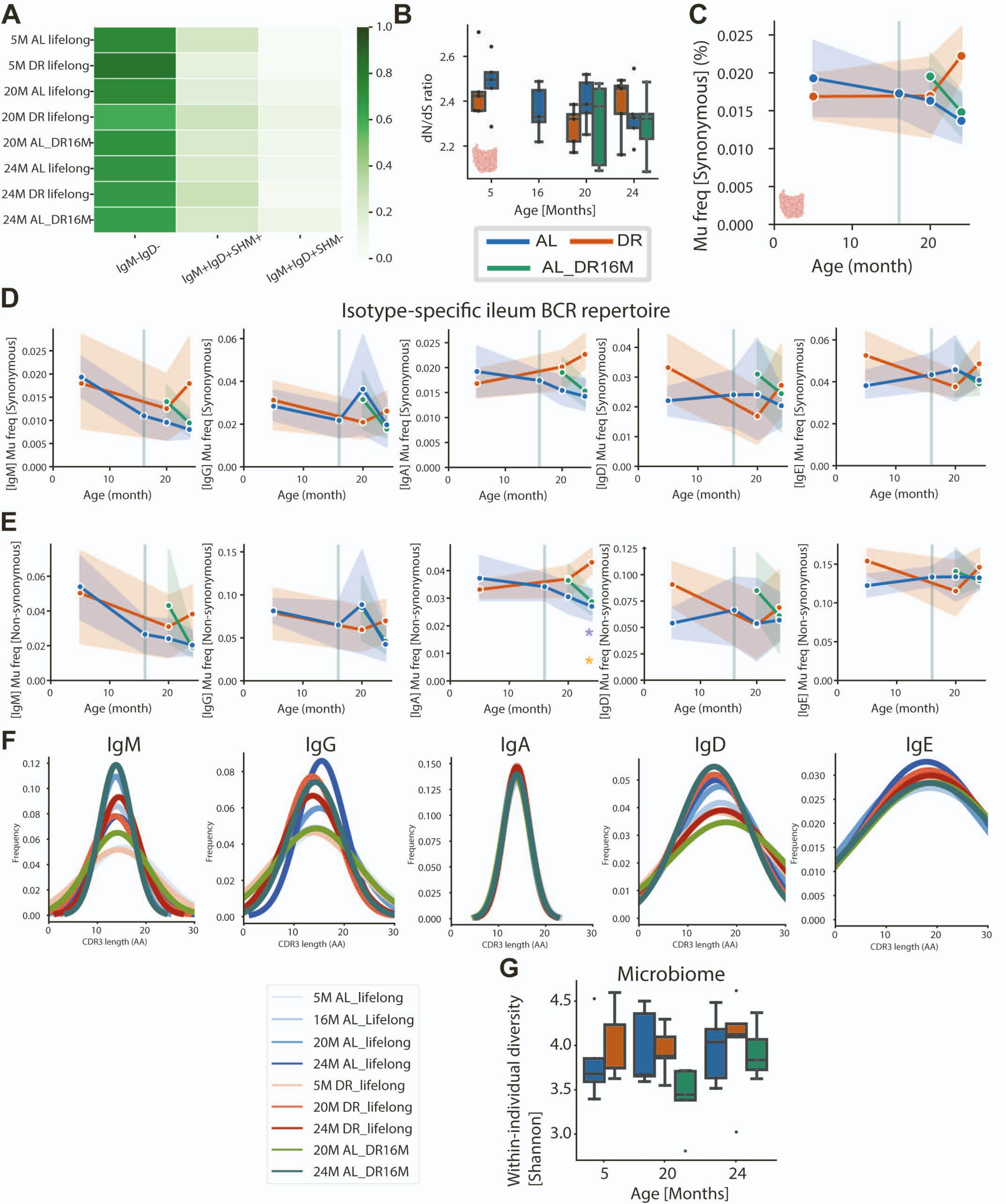

A
5M AL lifelong
5M DR lifelong
20M AL lifelong
20M DR lifelong
20M AL_DR16M
24M AL lifelong
24M DR lifelong
24M AL_DR16M
IgM-IgD-
IgM+IgD+SHM+
IgM+IgD+SHM-
B
dN/dS ratio
Age [Months]
AL
DR
AL_DR16M
C
Mu freq [Synonymous] (%)
Age (month)
D
Isotype-specific ileum BCR repertoire
[IgM] Mu freq [Synonymous]
[IgG] Mu freq [Synonymous]
[IgA] Mu freq [Synonymous]
[IgD] Mu freq [Synonymous]
[IgE] Mu freq [Synonymous]
Age (month)
E
[IgM] Mu freq [Non-synonymous]
[IgG] Mu freq [Non-synonymous]
[IgA] Mu freq [Non-synonymous]
[IgD] Mu freq [Non-synonymous]
[IgE] Mu freq [Non-synonymous]
Age (month)
F
IgM
IgG
IgA
IgD
IgE
Frequency
CDR3 length (AA)
5M AL_lifelong
16M AL_Lifelong
20M AL_lifelong
24M AL_lifelong
5M DR_lifelong
20M DR_lifelong
24M DR_lifelong
20M AL_DR16M
24M AL_DR16M
G
Microbiome
Within-individual diversity [Shannon]
Age [Months]

Figure S5. Isotype specific somatic hypermutation rate and CDR length of the ileum. Related to Figure 2.

A) Relative frequency of BCR clones in the ileum according to their class-switch status. **B)** Ratio of non-synonymous to synonymous mutations in ileum. **C)** Frequency of synonymous SHM in the ileum. **D)** Isotype-specific frequency of synonymous SHM in the ileum. Significant differences through age (linear regression): IgM (AL $p = 0.001$). Significant differences through age and diet (2-way ANOVA): IgA (Age * Diet $p = 0.015$)**. E)** Isotype-specific frequency of nonsynonymous SHM in the ileum. Significant differences through age (linear regression): IgM (AL $p = 0.006$), IgG (AL $p = 0.044$). Significant differences through age and diet (2-way ANOVA): IgA (Age * Diet $p = 0.006$). *p-value < 0.05, (Mann-Whitney U test) at individual timepoints represented in light purple for DR vs AL and orange for DR vs AL_DR16M. **F)** Isotype-specific CDR3-length distribution in the ileum. **G)** Within-individual diversity of caecal microbiome. n= 5 female mice per treatment. **B** and **G** boxes indicate quartiles, and whiskers the rest of the data distribution. **C**-**F** lines correspond to the mean, and shaded areas to 95% confidence intervals.

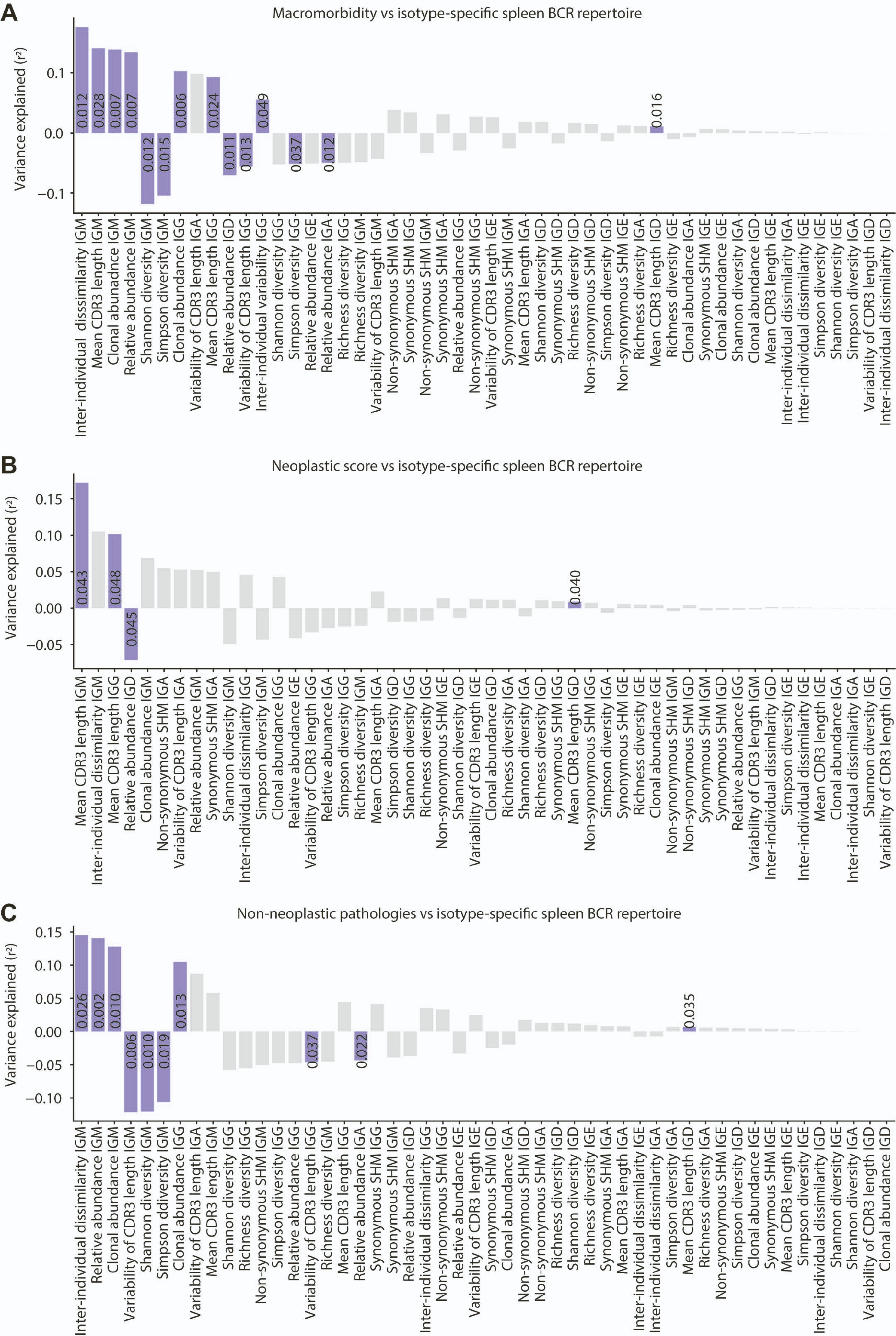
A
Macromorbidity vs isotype-specific spleen BCR repertoire
Variance explained (r²)
0.1
0.0
−0.1
0.012
0.028
0.007
0.007
0.012
0.015
0.006
0.024
0.011
0.013
0.049
0.037
0.012
0.016
Inter-individual disssimilarity IGM
Mean CDR3 length IGM
Clonal abunadnce IGM
Relative abundance IGM
Shannon diversity IGM
Simpson diversity IGM
Clonal abundance IGG
Variability of CDR3 length IGA
Mean CDR3 length IGG
Relative abundance IGD
Variability of CDR3 length IGG
Inter-individual variability IGG
Shannon diversity IGG
Simpson diversity IGG
Relative abundance IGE
Relative abundance IGA
Richness diversity IGG
Richness diversity IGM
Variability of CDR3 length IGM
Non-synonymous SHM IGA
Synonymous SHM IGG
Non-synonymous SHM IGM
Synonymous SHM IGA
Relative abundance IGG
Non-synonymous SHM IGG
Variability of CDR3 length IGE
Synonymous SHM IGM
Mean CDR3 length IGA
Shannon diversity IGD
Synonymous SHM IGD
Richness diversity IGD
Non-synonymous SHM IGD
Simpson diversity IGD
Non-synonymous SHM IGE
Richness diversity IGA
Mean CDR3 length IGD
Richness diversity IGE
Clonal abundance IGA
Synonymous SHM IGE
Clonal abundance IGE
Shannon diversity IGA
Clonal abundance IGD
Mean CDR3 length IGE
Inter-individual dissimilarity IGA
Inter-individual dissimilarity IGE
Simpson diversity IGE
Shannon diversity IGE
Simpson diversity IGA
Variability of CDR3 length IGD
Inter-individual dissimilarity IGD
B
Neoplastic score vs isotype-specific spleen BCR repertoire
Variance explained (r²)
0.15
0.10
0.05
0.00
−0.05
0.043
0.048
0.045
0.040
Mean CDR3 length IGM
Inter-individual dissimilarity IGM
Mean CDR3 length IGG
Relative abundance IGD
Clonal abundance IGM
Non-synonymous SHM IGA
Variability of CDR3 length IGA
Relative abundance IGM
Synonymous SHM IGA
Shannon diversity IGM
Inter-individual dissimilarity IGG
Simpson diversity IGM
Clonal abundance IGG
Relative abundance IGE
Variability of CDR3 length IGG
Relative abunance IGA
Simpson diversity IGG
Richness diversity IGM
Mean CDR3 length IGA
Simpson diversity IGD
Shannon diversity IGG
Richness diversity IGG
Non-synonymous SHM IGE
Shannon diversity IGD
Variability of CDR3 length IGE
Clonal abundance IGD
Richness diversity IGA
Shannon diversity IGA
Richness diversity IGD
Synonymous SHM IGG
Mean CDR3 length IGD
Non-synonymous SHM IGG
Simpson diversity IGA
Synonymous SHM IGE
Richness diversity IGE
Clonal abundance IGE
Non-synonymous SHM IGM
Non-synonymous SHM IGD
Synonymous SHM IGM
Synonymous SHM IGD
Relative abundance IGG
Variability of CDR3 length IGM
Inter-individual dissimilarity IGD
Simpson diversity IGE
Inter-individual dissimilarity IGE
Mean CDR3 length IGE
Clonal abundance IGA
Inter-individual dissimilarity IGA
Shannon diversity IGE
Variability of CDR3 length IGD
C
Non-neoplastic pathologies vs isotype-specific spleen BCR repertoire
Variance explained (r²)
0.15
0.10
0.05
0.00
−0.05
−0.10
0.026
0.002
0.010
0.006
0.010
0.019
0.013
0.037
0.022
0.035
Inter-individual dissimilarity IGM
Relative abundance IGM
Clonal abundance IGM
Variability of CDR3 length IGM
Shannon diversity IGM
Simpson ddiversity IGM
Clonal abundance IGG
Variability of CDR3 length IGA
Mean CDR3 length IGM
Shannon diversity IGG
Richness diversity IGG
Non-synonymous SHM IGM
Simpson diversity IGG
Relative abundance IGG
Variability of CDR3 length IGG
Richness diversity IGM
Mean CDR3 length IGG
Relative abundance IGA
Synonymous SHM IGG
Synonymous SHM IGM
Relative abundance IGD
Inter-individual dissimilarity IGG
Non-synonymous SHM IGG
Relative abundance IGE
Variability of CDR3 length IGE
Synonymous SHM IGD
Clonal abundance IGA
Non-synonymous SHM IGD
Non-synonymous SHM IGA
Richness diversity IGD
Shannon diversity IGD
Richness diversity IGE
Synonymous SHM IGA
Mean CDR3 length IGA
Inter-individual dissimilarity IGE
Inter-individual dissimilarity IGA
Simpson diversity IGA
Mean CDR3 length IGD
Richness diversity IGA
Non-synonymous SHM IGE
Simpson diversity IGD
Clonal abundance IGE
Synonymous SHM IGE
Mean CDR3 length IGE
Simpson diversity IGE
Inter-individual dissimilarity IGD
Shannon diversity IGE
Shannon diversity IGA
Variability of CDR3 length IGD
Clonal abundance IGD

Figure S6. Correlation of isotype-specific BCR metrics in the spleen with morbidity and pathology. Related to Figure 3.

A) Macromorbidity, **B)** neoplastic score and **C)** non-neoplastic pathology burden. Coloured bars indicate significant association (Spearman correlation $p < 0.05$). n= 5 female mice per treatment.

OLIGONUCLEOTIDES	SOURCE
SmartNNNa: AAGCAGUGGTAUCAACGCAGAGUNNNNUNNNNUNNNNUCTTrGrGrGrG	[1]
M1SS (mix): AAGCAGTGGTATCAACGCA	[1]
mIGA_r1: CCAGGTCACATTCATCGTG	[1]
mIGA_r2: ATTGGGCAGCCCTGATTTCAGTGGGTAGATGGTG	[1]
mIGD_r1: GCCATTTCTCATTTCAGAGG	[1]
mIGD_r2: ATTGGGCAGCCCTGATTCTCTGAGAGGAGGAAC	[1]
mIGE_r1: GTTCACAGTGCTCATGTTC	[1]
mIGE_r2: ATTGGGCAGCCCTGATTAAGGGGTAGAGCTGAG	[1]
mIGG3_r1: GTACAGTCACCAAGCTGCT	[1]
mIGG3_r2: ATTGGGCAGCCCTGATTAAGGGATAGACAGATG	[1]
mIGG12_r1: KKACAGTCACTGAGCTGCT	[1]
mIGG12_r2: ATTGGGCAGCCCTGATTAGTGGATAGACMGATG	[1]
mIGM_r1: CTGGATGACTTCAGTGTTGT	[1]
mIGM_r2: ATTGGGCAGCCCTGATTGGGGGAAGACATTTGG	[1]
M1S: (N)4–6(XXXXX)CAGTGGTATCAACGCAGAG	[1]
Z: (N)4-6(XXXXX)ATTGGGCAGCCCTGATT	[1]

V4-515F: ACACTCTTTCCCTACACGACGCTCTTCCGATCTGTGYCAGCMGCCGCGGTAA	2
V4-806R: GTGACTGGAGTTCAGACGTGTGCTCTTCCGATCTGGACTACNVGGGTWTCTAAT	2

Table S1: Oligonucleotides for B cell receptor repertoire and 16S rRNA sequencing.

References

1. Turchaninova, M.A., Davydov, A., Britanova, O.V., Shugay, M., Bikos, V., Egorov, E.S., Kirgizova, V.I., Merzlyak, E.M., Staroverov, D.B., Bolotin, D.A., et al. (2016). High-quality full-length immunoglobulin profiling with unique molecular barcoding. Nat. Protoc. 11, 1599–1616. 10.1038/nprot.2016.093.

2. Caporaso, J.G., Lauber, C.L., Walters, W.A., Berg-Lyons, D., Lozupone, C.A., Turnbaugh, P.J., Fierer, N., and Knight, R. (2011). Global patterns of 16S rRNA diversity at a depth of millions of sequences per sample. Proc. Natl. Acad. Sci. U. S. A. 108 Suppl 1, 4516–4522. 10.1073/pnas.1000080107.
